# The medial orbitofrontal cortex mediates neuropathic pain and anxiodepressive-like behaviors via two distinct pathways

**DOI:** 10.1186/s10194-026-02335-w

**Published:** 2026-03-18

**Authors:** Zhixiao Li, Tianen Si, Liangliang Gao, Yijie Meng, Xinyu Su, Liang Tao, Jingjing Zhang, Zhonghui Hu, Yao Ge, Xianglei Meng, Jing Cao

**Affiliations:** 1 Department of Anatomy, School of Basic Medical Sciences, Zhengzhou University, Zhengzhou, Henan China; 2 Emergency Department, The First Affiliated Hospital of Zhengzhou University, Zhengzhou, Henan China

**Keywords:** Neuropathic pain, Medial orbitofrontal cortex (mOFC), Anxiety and depression, Basolateral amygdala (BLA), Mediodorsal thalamus (MD)

## Abstract

**Background:**

Chronic neuropathic pain is frequently accompanied by anxiety and depression, yet the cortical circuit mechanisms underlying this comorbidity remain unclear. The medial orbitofrontal cortex (mOFC) is a key cortical region involved in emotional regulation and valuation, and clinical imaging studies have reported altered mOFC activity in patients with chronic pain. However, it remains unclear how mOFC neuronal activity contributes to both the pain hypersensitivity and anxiodepressive-like behaviors associated with neuropathic pain. This study aimed to determine the pathway-specific roles of mOFC glutamatergic neurons in chronic pain and comorbid affective disturbances.

**Methods:**

Neuropathic pain was induced by chronic constriction injury (CCI) of the sciatic nerve. Pain sensitivity and anxiodepressive-like behaviors were assessed using von Frey, Hargreaves, open field, elevated plus maze, tail suspension, and forced swim tests. Electrophysiological recording and fiber photometry were used to monitor neuronal activity. Neuronal projection tracing identified projection patterns of mOFC glutamatergic neurons to mediodorsal thalamus (MD) and basolateral amygdala (BLA). Chemogenetic and optogenetic manipulations were applied to selectively modulate the mOFC^CaMKIIα^-MD and mOFC^CaMKIIα^-BLA pathways.

**Results:**

The mOFC glutamatergic neurons are activated in neuropathic pain mice accompanied by anxiodepressive-like phenotypes. Chemogenetic and optogenetic inhibition of mOFC glutamatergic neurons attenuates both pain hypersensitivity and anxiodepressive-like behaviors after nerve injury. Retrograde labeling result revealed two non-overlapping mOFC^CaMKIIα^ neurons projecting to the MD and BLA, respectively. Selective inhibition of mOFC^CaMKIIα^-MD pathway leads to amelioration of CCI-induced pain hypersensitivity. While selective inhibition of mOFC^CaMKIIα^-BLA pathway leads to amelioration of CCI-induced anxiodepressive-like behaviors.

**Conclusions:**

The present study has revealed that the critical involvement of mOFC glutamatergic neurons in the comorbidity of chronic pain and affective disturbances. Inhibition of mOFC glutamatergic neurons attenuates both pain hypersensitivity and anxiodepressive-like behaviors after nerve injury. The discrete mOFC^CaMKIIα^-MD and mOFC^CaMKIIα^-BLA pathway independently control the sensory and affective components of neuropathic pain.

**Graphical Abstract:**

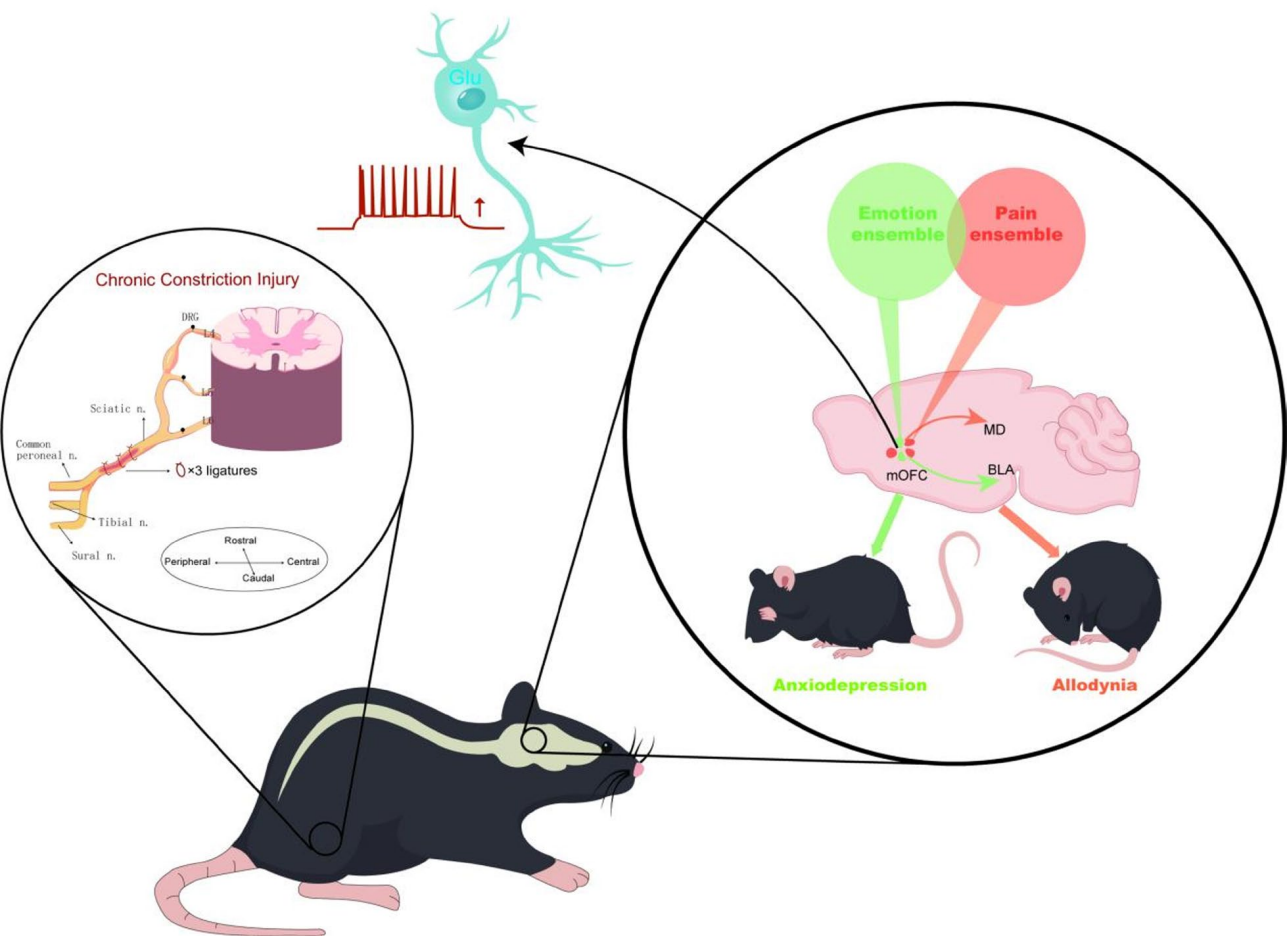

**Supplementary Information:**

The online version contains supplementary material available at 10.1186/s10194-026-02335-w.

## Introduction

Neuropathic pain can arise from a variety of etiologies and is clinically characterized by spontaneous pain and hyperalgesia, with a prevalence estimated to reach approximately 10% of the global population [1, 2]. Notably, approximately 60% of Patients suffering from chronic neuropathic pain also exhibit depression, anxiety, underscoring the strong bidirectional relationship between pain and affective disturbances [3, 4]. Growing clinical evidence suggests that the limited or short-lasting efficacy of neuropathic pain treatments is strongly influenced by psychological factors, particularly psychiatric comorbidities that substantially impair quality of life [5, 6]. Therefore, finding effective therapeutic strategies for pain and psychiatric comorbidities poses a major research challenge and promises significant medical and social impacts.

Increasing evidence indicates that multiple cortical regions, such as the anterior cingulate cortex (ACC), prefrontal cortex (PFC), and orbitofrontal cortex (OFC), contribute to the integration of sensory and emotional dimensions of pain. These cortical areas interact extensively with subcortical nuclei, including the mediodorsal thalamus (MD), ventral tegmental area (VTA), basolateral amygdala (BLA), periaqueductal gray (PAG), and nucleus accumbens (NAc), forming neural networks that jointly regulate chronic pain and affective disturbances [7–11]. In addition, several studies have shown that tricyclic antidepressants can effectively alleviate chronic neuropathic pain [12–14]. This therapeutic overlap may reflect shared neurobiological mechanisms between chronic pain and affective disorders, including common alterations in neural circuit activity, synaptic plasticity, and neuroinflammatory signaling [15, 16].

As a key component of the cortico–limbic system, the orbitofrontal cortex (OFC) plays an essential role in emotion regulation, reward processing, and decision-making [17–19]. Recent clinical studies have shown that intracranial OFC activity is closely associated with chronic pain states [20], and OFC dysfunction has been implicated in the development of anxiety and depression [21, 22]. The OFC contains several distinct subregions that differ in their functional roles [23–26]. The ventrolateral OFC (vlOFC) has been identified as part of a descending analgesic pathway, where activation of glutamatergic neurons in the vlOFC relieves hypersensitivity after peripheral nerve injury via activation of the vlOFC-vlPAG circuit [27]. In contrast, accumulating evidence indicates that the medial orbitofrontal cortex (mOFC) is more strongly associated with emotional regulation and chronic pain. Enhanced θ- and α-band oscillations in the mOFC have been linked to local hypersensitivity in chronic pain conditions [28, 29], and elevated mOFC blood flow has been reported in patients with depression [30, 31]. Despite this evidence, whether and how the neural substrates of the mOFC modulate neuropathic pain and anxiodepressive-like behaviors remains largely unknown. Here, by using the electrophysiological recordings, optical fiber photometry recordings in free-moving mice, and pathway-specific optogenetic and chemogenetic manipulations, we identify two non-overlapping neuronal populations in the mOFC respectively modulate neuropathic pain and anxiodepressive-like behaviors after nerve injury by projecting to the MD and BLA.

## Result

### The mOFC glutamatergic neurons are activated in neuropathic pain mice accompanied by anxiodepressive-like phenotypes

**Fig. 1:**
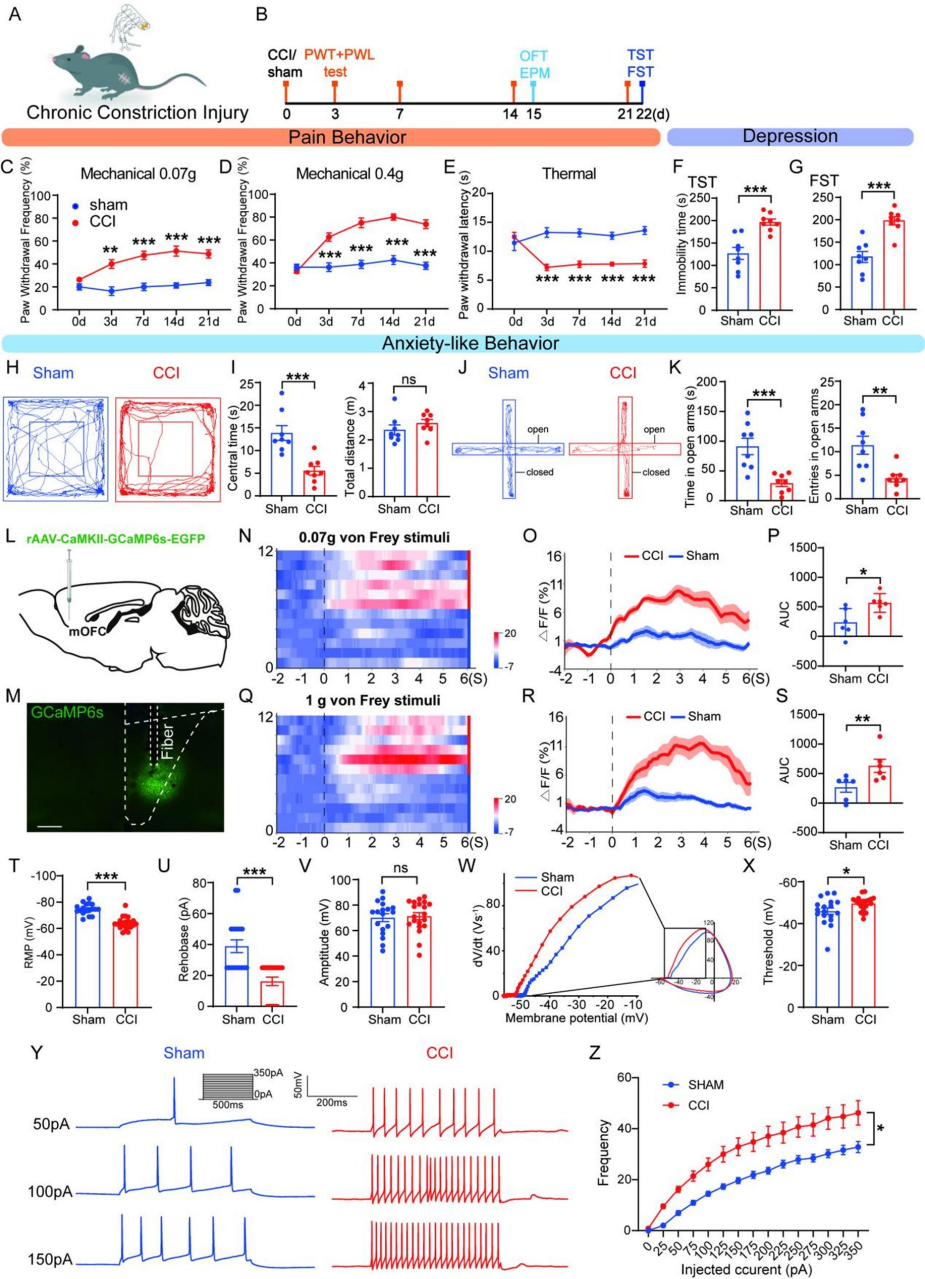
Increased activity of mOFC glutamatergic neurons in neuropathic pain mice comorbid with anxiodepressive-like phenotypes. (**A**) Chronic Constriction Injury (CCI) model schematic. (**B**) Experimental schedule of the CCI surgery and behavioral tests. (**C**-**E**) Mechanical PWF (**C**, **D**) and thermal PWL (**G**) of Sham mice and CCI mice (two-way ANOVA with Sidak post hoc test, *n* = 8/group). (**F**, **G**) Immobility time in TST (**F**) and FST (unpaired t-test, *n* = 8/group). (**H**-**K**) The example traces of OFT (**H**) and EPM (**J**) from Sham and CCI mice 2 weeks after modeling. Quantification of central time (**I** left, unpaired t-test, *n* = 8/group), and the total distance (**I** right, Mann-Whitney test, *n* = 8/group) in the OFT from Sham and CCI mice. Quantification of open arm time (**K** left) and entries (**K** right) from Sham and CCI mice (unpaired t-test, *n* = 8/group). (**L**) Schematic of in vivo calcium imaging experimental design. (**M**) Representative images of the virus injection site of the mOFC (AP = + 2.45 mm). Scale bar: 100 μm (**N**-**S**) Heatmaps and Mean Ca²⁺ signal traces demonstrating increased Ca²⁺ responses in CCI mice compared to Sham controls during 0.07 g (**N**, **O**) and 1 g (**Q**, **R**) mechanical stimulation. Quantification of area under the curve (AUC, 0–6 s) of fluorescence changes during 0.07 g (**P**, unpaired t-test) and 1 g (**S**, Mann-Whitney test) mechanical stimulation (*n* = 6 events from 6 mice/group). (**T**-**V**) RMP (**T**, unpaired t-test), Rheobase (**U**, Mann-Whitney test), and Amplitude (**V**, Mann-Whitney test) of mOFC CaMKIIα^+^ neurons from Sham and CCI mice (*n* = 18 or 20 from 5 mice/group). (**W**) Phase plane plot (Vm versus dV/dt) for action potentials from Sham and CCI. Voltage threshold was defined as dV/dt = 20 mV/ms^− 1^. The region around the threshold has been magnified for clarity. (**X**) Threshold of mOFC CaMKIIα^+^ neurons from Sham and CCI mice (unpaired t-test, *n* = 18 or 20 from 5 mice/group). (**Y**) Representative firing traces of evoked APs. (**Z**) The analysis of AP frequency against the injecting currents (two-way ANOVA with Sidak post hoc test, *n* = 18 or 20/group). Data were presented as the mean ± SEM. *P < 0.05, **P < 0.01, ***P < 0.001, ns = no significant

Neuropathic pain was induced in mice using the chronic constriction injury (CCI) model (Fig. 1A). Sensory and emotional behaviors were evaluated after surgery following the timeline (Fig. 1B). No statistical differences in paw withdrawal frequency (PWF) or paw withdrawal latency (PWL) were observed between the sham and CCI groups before surgery. From day 3 to day 21 post-surgery, PWF (0.07 g and 0.4 g) was significantly increased (Fig. 1C, D), whereas PWL significantly decreased (Fig. 1E) compared with the Sham group (*p* < 0.001), indicating that CCI induced chronic neuropathic pain. Since anxiety and depression are frequently comorbid with chronic pain in clinical patients [32], we also conducted anxiety-related behavioral tests using the open field test (OFT) and elevated plus maze test (EPM), and depression-related behavioral tests using the tail suspension test (TST) and forced swimming test (FST). These tests were performed from day 14 after CCI, a time point when anxiodepressive-like behaviors are stably manifested according to previous studies [33, 34]. The CCI group spent significantly less time in the center area of the open field, with no significant difference in the total distance traveled (Fig. 1H, I), and they also spent less time and fewer entries in the open arms of EPM compared to the Sham group after day 14 post-surgery (Fig. 1J, K). Furthermore, the CCI group exhibited significantly higher immobility time in TST and FST compared to the Sham group (Fig. 1F, G) after day 21 post-surgery. These results suggested that CCI induced both neuropathic pain and anxiodepressive-like behaviors.

**Fig. 2:**
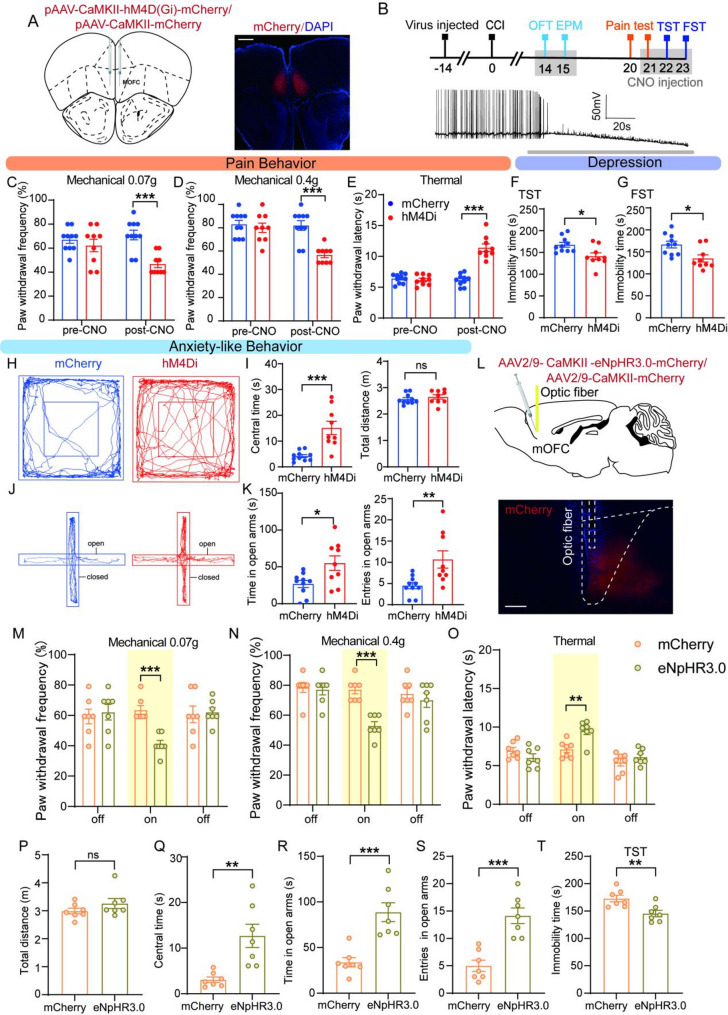
Inhibition of mOFC glutamatergic neurons ameliorated pain hypersensitivity and anxiodepressive-like behaviors in CCI mice. (**A**) Schematic diagram of stereotaxic delivery of AAV-CaMKII-hM4D(Gi)-mCherry (left) and representative images indicating the injection site was mOFC (right, AP = + 2.45 mm). Scale bar: 100 μm (**B**) Behavioral tests timeline (up) and examples showing that the effects of bath CNO (500 nM) on mCherry^+^ neurons firing in mOFC slices from mice injected with AAV-CaMKII-hM4D(Gi)- mCherry (down). (**C**-**E**) Effect of chemogenetic inhibition of mOFC glutamatergic neurons on pain behavior in 0.07 g (**C**), 0.4 g (**D**) von Frey and Hargreaves tests (**E**) (two-way ANOVA with Sidak post hoc test, *n* = 9/10 mice/group). (**F**-**K**) Effect of chemogenetic inhibition of mOFC glutamatergic neurons on anxiodepressive-like behavior in TST (**F**, unpaired t-test), FST (**G**, unpaired t-test), OFT (**H**, **I**), and EPM (**J**, **K**) (unpaired t-test, *n* = 9/10 mice/group). (**L**) Schematic and photomicrograph showing the site of optical fiber implantation and AAV-CaMKII-eNpHR3.0 -mCherry injection into the mOFC (AP = + 2.45 mm). Scale bar: 200 μm. (**M**-**O**) Effect of optogenetic inhibition of mOFC glutamatergic neurons on pain behavior in 0.07 g (**M**), 0.4 g (**N**) von Frey and Hargreaves tests (**O**) (two-way ANOVA with Sidak post hoc test, *n* = 7 mice/group). (**P**-**T**) Effect of optogenetic inhibition of mOFC glutamatergic neurons on anxiodepressive-like behavior in OFT (**P**, **Q**), EPM (**R**, **S**), and TST (**F**) (unpaired t-test, *n* = 7 mice/group). Data were presented as the mean ± SEM. *P < 0.05, **P < 0.01, ***P < 0.001, ns = no significant

There is evidence that mOFC is a key region implicated in both pain and mood regulation [10, 35, 36]. To determine whether the mOFC is involved in the mechanism of neuropathic pain–induced anxiodepressive-like behaviors, we assessed the neuronal excitability of MOFC glutamatergic neurons. We injected AAV-CaMKII-GCaMp6s-EGFP into the mOFC of C57 mice; viral expression was confirmed after 21 days post-CCI (Fig. 1L, M). Functional assessment revealed that CCI induced enhanced calcium responses of MOFC glutamatergic neurons to both non-noxious (0.07 g, Fig. 1, N-P) and noxious (1 g, Fig. 1, Q-S) von Frey stimulation compared with Sham controls. We further assessed the MOFC glutamatergic neuronal excitability of *CaMKII-Cre*,* Ai14* mice using the whole-cell patch-clamp recording. Specifically, these neurons exhibited a depolarized resting membrane potential (RMP) (Fig. 2T), reduced rheobase current (Fig. 2U), and a lower action potential (AP) threshold (Fig. 2W, X) following nerve injury. Moreover, the CCI group displayed an increased number of spikes in response to depolarizing current injections (Fig. 2Y, Z), while AP amplitude remained unchanged compared with Sham controls (Fig. 2V). These findings indicate that CCI induced anxiodepressive-like behaviors accompanied by increased mOFC glutamatergic neuronal activity. However, whether the activation of glutamatergic neurons is a key driver of comorbid pain hypersensitivity and anxiodepressive behaviors in CCI mice remains to be determined.

### Chemogenetic and optogenetic inhibition of mOFC glutamatergic neurons attenuates pain hypersensitivity and anxiodepressive-like behaviors after nerve injury

To explore the role of the mOFC glutamatergic neurons in neuropathic pain and anxiodepressive-like behaviors, we inhibited MOFC glutamatergic neurons by chemogenetic manipulation. The adeno-associated virus–encoding engineered Gi-coupled hM4D receptor (AAV-CaMKII-hM4Di-mCherry) or AAV-CaMKII-mCherry (control) was bilaterally injected into the mOFC. Histological analysis confirmed hM4Di-mCherry expression in mOFC neurons (Fig. 2A). The mice underwent sham surgery or nerve injury for 2 weeks after the injections (Fig. 2B). The efficacy of hM4Di-mediated excitation was confirmed by whole-cell patch clamp recordings in mOFC slices. Action potential (AP) firing was inhibited by CNO (500 nM) in mOFC neurons expressing hM4Di (Fig. 2B).

The mice received an intraperitoneal injection of CNO (2 mg/kg) 1 h before the behavioral tests to achieve specific inhibition of mOFC neurons expressing hM4D receptor. Behavioral data revealed that CNO injection decreased the mechanical withdrawal frequency and increased the thermal withdrawal latencies in CCI mice (Fig. 2C-E). Antidepressant-like behaviors were induced by inhibiting mOFC glutamatergic neurons in CCI mice. Both the immobile duration in TST and FST were lower in the hM4Di group than the mCherry group (Fig. 2F, G). Consistently, the hM4Di group exhibited typical antianxiety-like behaviors, marked by more central time in the OFT test, as well as more open arm time and open arm entries in the EPM test (Fig. 2, H-K). There was no difference between the two groups in total travel distance in the OFT, indicating that the phenotypic differences were not due to alterations in locomotor activity (Fig. 2I). In addition, inhibition of mOFC glutamatergic neurons in normal mice produced only a significant increase in open-arm entries in the elevated plus maze, while other pain-related and anxiodepressive-like behaviors were not significantly altered (Fig. S1A-K).

As a complementary approach, we injected AAV2-CaMKII-eNpHR3.0-mCherry into the mOFC, resulting in robust mCherry expression at the injection site (Fig. 2L). An optic fiber was implanted above the mOFC to deliver yellow laser light for activation of eNpHR3.0. Consistent with the chemogenetic results, optogenetic inhibition of mOFC glutamatergic neurons significantly attenuated mechanical and thermal hyperalgesia in CCI mice (Fig. 2M-O) and alleviated anxiodepressive-like behaviors (Fig. 2Q-T). Moreover, optogenetic stimulation did not affect general locomotor activity (Fig. 2P). Together, these findings demonstrate that both chemogenetic and optogenetic inhibition of mOFC glutamatergic neurons reduce pain hypersensitivity and anxiodepressive-like behaviors following nerve injury.

### Chemogenetic and optogenetic activation of mOFC glutamatergic neurons induces pain and anxiodepressive-like behaviors

**Fig. 3:**
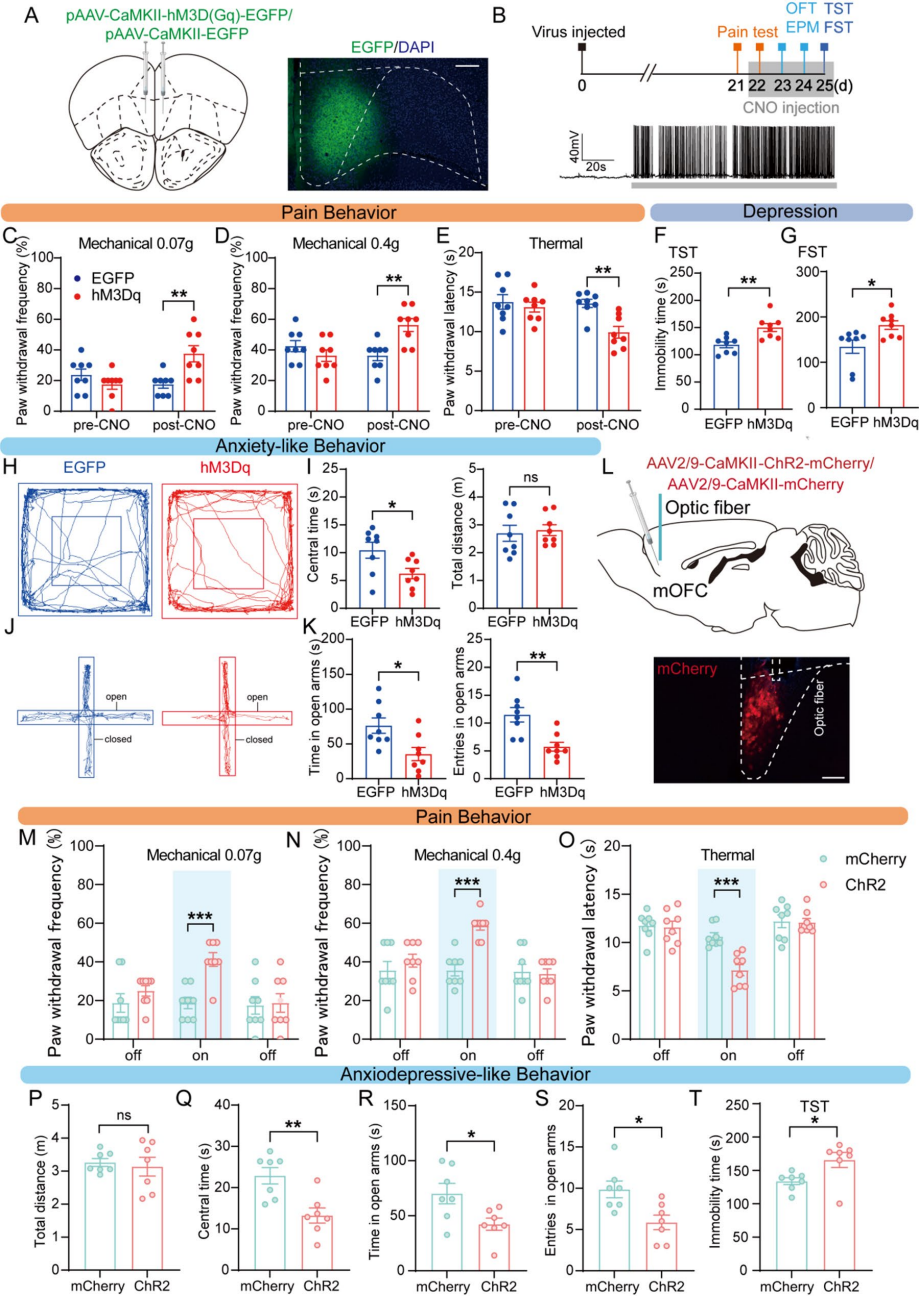
Activation of mOFC glutamatergic neurons induces pain and anxiodepressive-like behaviors. **(A)** Schematic diagram of stereotaxic delivery of AAV-CaMKII-hM3D(Gq)-EGFP (left) and representative images indicating the injection site was mOFC (right, AP = + 2.45 mm). Scale bar: 100 μm **(B)** Behavioral tests timeline (up) and examples showing that the effects of bath CNO (500 nM) on EGFP^+^ neurons firing in mOFC slices from mice injected with AAV-CaMKIIα-hM3D(Gq)- EGFP (down). **(C-E)** Effect of chemogenetic activation of mOFC glutamatergic neurons on pain behavior in 0.07 g (**C**), 0.4 g (**D**) von Frey and Hargreaves tests (**E**) (two-way ANOVA with Sidak post hoc test, *n* = 8 mice/group). **(F-K)** Effect of chemogenetic activation of mOFC glutamatergic neurons on anxiodepressive-like behavior in TST (**F**, unpaired t-test), FST (**G**, Mann-Whitney test), OFT (**H**, **I**), and EPM (**J**, **K**) (unpaired t-test, *n* = 8 mice/group). **(L)** Schematic and photomicrograph showing the site of optical fiber implantation and AAV-CaMKII-ChR2-mCherry injection into the mOFC (AP = + 2.45 mm). Scale bar: 100 μm. **(M-O)** Effect of optogenetic activation of mOFC glutamatergic neurons on pain behavior in 0.07 g (**M**), 0.4 g (**N**) von Frey and Hargreaves tests (**O**) (two-way ANOVA with Sidak post hoc test, *n* = 8 mice/group). **(P-T)** Effect of optogenetic activation of mOFC glutamatergic neurons on anxiodepressive-like behavior in OFT (**P**, **Q**, unpaired t-test), EPM (**R**, **S**, unpaired t-test), and TST (**F**, Mann-Whitney test) (*n* = 7 mice/group). Data were presented as the mean ± SEM. *P < 0.05, **P < 0.01, ***P < 0.001, ns = no significant

To test whether activation of mOFC glutamatergic neurons induced pain hypersensitivity and anxiodepressive-like behaviors in mice. We next activated mOFC glutamatergic neurons chemogenetically with bilateral injection of AAV-encoding engineered Gq-coupled hM3D receptor (AAV-CaMKII-hM3Dq-EGFP) or AAV-CaMKII-EGFP (control) into the mOFC in naïve mice (Fig. 3A). Whole-cell patch clamp recordings in mOFC slices showed that CNO perfusion induced AP firing, confirming the hM3Dq-mediated activation effect on mOFC glutamatergic neurons (Fig. 3B).

Behavioral testing was performed three weeks after viral delivery. Following CNO administration, mice expressing hM3Dq-EGFP in mOFC glutamatergic neurons exhibited a higher mechanical withdrawal frequency and shorter thermal withdrawal latencies compared to EGFP controls, indicating enhanced pain sensitivity (Fig. 2C–E). Then, we tested the effects of specific activation of mOFC glutamatergic neurons on anxiodepressive-like behaviors. Activation of mOFC glutamatergic neurons also increased immobility duration in the TST and FST (Fig. 2F–G) and reduced center time in the OFT, as well as open-arm time and entries in the EPM (Fig. 2H–K). Importantly, total travel distance in the OFT did not differ between groups, suggesting that these phenotypic changes were not due to altered locomotor activity. In addition, our results found that activating mOFC glutamatergic neurons failed to induce significant changes in pain or anxiodepressive-like behaviors in CCI mice (Fig. S2A–K). This lack of effect is likely due to a ceiling effect, meaning that pain hypersensitivity and negative affect were already maximally expressed after CCI, leaving no room for further exacerbation.

To complement these findings, we injected AAV2-CaMKII-ChR2-mCherry into the mOFC, resulting in robust mCherry expression at the injection site (Fig. 3L). An optic fiber was implanted above the mOFC to deliver blue laser light for activation of ChR2. Consistent with the chemogenetic results, optogenetic activation of mOFC glutamatergic neurons induced mechanical and thermal hyperalgesia in Naive mice (Fig. 3M–O) and anxiodepressive-like behaviors (Fig. 3Q–T). Moreover, optogenetic stimulation did not affect general locomotor activity (Fig. 3P). Together, these results demonstrate that both chemogenetic and optogenetic activation of mOFC glutamatergic neurons can induce both pain hypersensitivity and anxiodepressive-like behaviors in Naïve mice.

### Differential projection patterns of mOFC glutamatergic neurons to MD and BLA

**Fig. 4:**
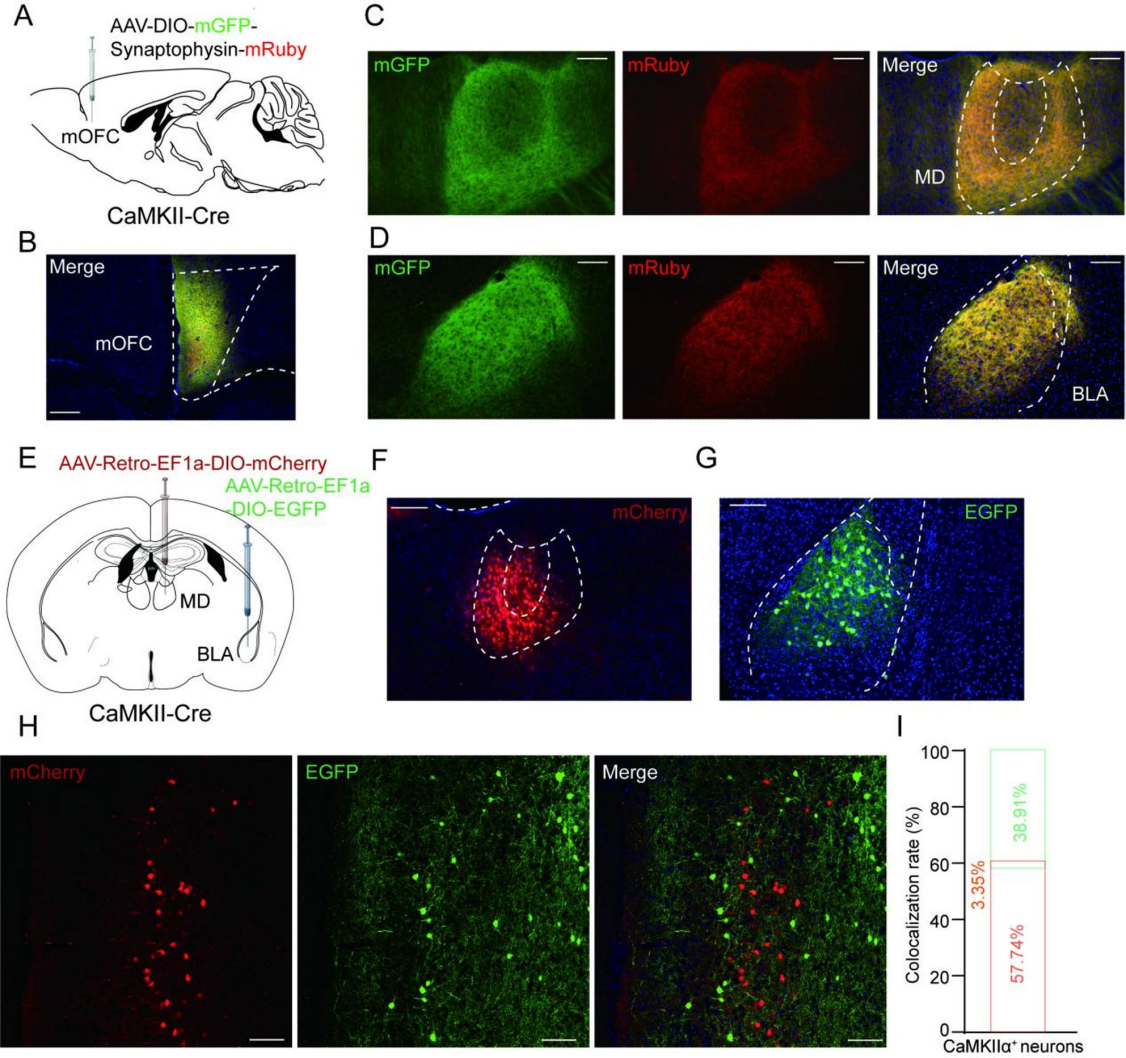
Distinct ensembles of mOFC glutamatergic neurons exhibit segregated projections to BLA and MD. **(A)** Schematic diagram of stereotaxic delivery of AAV-DIO-mGFP-2 A- Synaptophysin-mRuby in mOFC of *CaMKII-Cre* mice. **(B)** The expression of mGFP and mRuby in the mOFC (AP = + 2.45 mm). Scale bar: 100 μm. **(C**,** D)** Representative images showing that the mOFC CaMKIIα^+^ neurons project to MD (mediodorsal thalamus, **C**, AP = − 1.23 mm) and BLA (basolateral amygdala, **D**, AP = − 1.31 mm). Scale bar: 50 μm. **(E)** Diagram of retrograde tracing approach in *CaMKII-Cre* mice. **(F)** Validation of the injection site of AAV-Retro-EF1a-DIO-mCherry in MD (AP = − 1.23 mm). **(G)** Validation of the injection site of AAV-Retro-EF1a-DIO -EGFP in BLA (AP = − 1.31 mm). Scale bar: 100 μm. **(H)** Representative images of the mOFC CaMKIIα^+^ neurons projecting to the MD (red) and BLA (green). Scale bar: 50 μm (AP = + 2.30 mm). **(I)** Quantification of the colocalization rate of retrograde tracing of MD neurons labeled by mCherry and BLA neurons labeled by EGFP. *n* = 3 mice

The optogenetic and chemogenetic manipulations described above induce global alterations in mOFC glutamatergic output, potentially influencing projections to multiple downstream targets. To further investigate the circuit mechanisms by which mOFC glutamatergic neurons mediate comorbidity of neuropathic pain and anxiodepressive behaviors, we injected Cre-dependent AAVs expressing membrane-bound GFP (mGFP, for labeling axons) and synaptophysin-mRuby (for labeling putative presynaptic sites) into the mOFC of *CaMKII-Cre* mice (Fig. 4A). Viral expression of mOFC was confirmed after three weeks post virus injection (Fig. 4B). Brain sections revealed that the mOFC projects to multiple brain regions including ACC and NAc, among others (Fig. S3), with the most clear colocalization of mGFP-labeled green axonal fibers and mRuby-positive red presynaptic puncta in the MD and BLA (Fig. 4C, D).

To determine projection patterns of mOFC neurons projecting to the MD and BLA, we injected retrograde AAV-Retro-hSyn-mCherry into the MD and AAV-Retro-hSyn-EGFP into the BLA of C57 mice (Fig. S4A). Viral expression sites in MD and BLA were confirmed after three weeks post-virus injection (Fig. S4B, C). The brain sections of mOFC revealed non-overlapping populations of retrogradely labeled neurons, indicating that mOFC projections to the MD and BLA arise from largely distinct neuronal ensembles (Fig. S4D, E). To further validate this projection, we injected Cre-dependent AAV-Retro-EF1a-DIO-mCherry into the MD and AAV-Retro-EF1a-DIO-EGFP into the BLA of *CaMKII-Cre* mice (Fig. 4E). Three weeks after virus injection, we confirmed robust and spatially confined viral expression at both target sites (Fig. 4F, G). The mOFC brain sections also revealed non-overlapping populations of retrogradely labeled neurons, suggesting that CaMKIIα⁺ neurons in the mOFC exhibit differential projection patterns to the MD and BLA (Fig. 4H, I). Given these anatomically distinct projection patterns, we next selectively manipulated each pathway to examine its specific contribution to neuropathic pain-related behaviors.

### Selective inhibition of mOFC^CaMKIIα^-MD pathway leads to amelioration of CCI-induced pain hypersensitivity

**Fig. 5:**
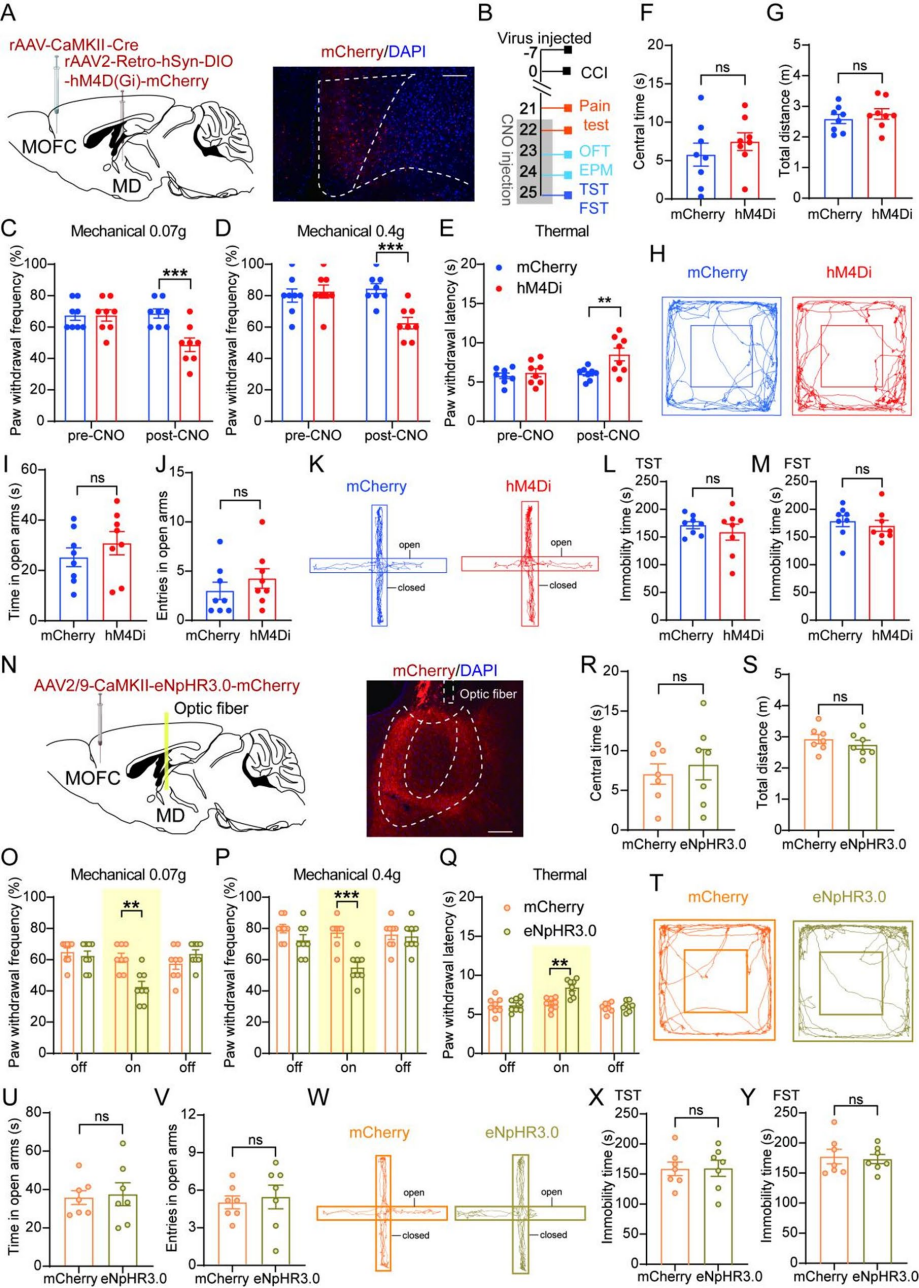
Chemogenetic and optogenetic inhibition of mOFC^CaMKIIα^-MD projections alleviates CCI-induced pain behaviors. **(A)** Schematic of experimental design (left) and expression of hM4D(Gi)-mCherry in the mOFC (right, AP = + 2.45 mm). Scale bar: 100 μm. **(B)** Viral injection and behavioral tests timeline. **(C-E)** Effect of chemogenetic inhibition of mOFC^CaMKIIα^-MD pathway neurons on pain behavior in 0.07 g (**C**), 0.4 g (**D**) von Frey and Hargreaves tests (**E**) (two-way ANOVA with Sidak post hoc test, *n* = 8/group). **(F-H)** Quantification of the central time (**F**) and total distance (**G**) and the example traces (**H**) in the OFT from the mCherry and hM4D(Gi)-mCherry group (unpaired t-test, *n* = 8/group). **(I-K)** Quantification of the time (**I**) and entries (**J**) in open arms and the example traces (**K**) in the EPM from the mCherry and hM4D(Gi)-mCherry group (unpaired t-test, *n* = 8/group). **(L**,** M)** Immobility time in TST (**L**) and FST (**M**) from the mCherry and hM4D(Gi)-mCherry group (unpaired t-test, *n* = 8/group). **(N)** Schematic of experimental design (left) and the axon terminals expression of eNpHR3.0-mCherry and optic fiber implantation in the MD (right, AP = − 1.23 mm). Scale bar: 100 μm. **(O-Q)** Effect of optogenetic inhibition of mOFC^CaMKIIα^-MD pathway neurons on pain behavior in 0.07 g (**O**), 0.4 g (**P**) von Frey and Hargreaves tests (**Q**) (two-way ANOVA with Sidak post hoc test, *n* = 8/group). **(R-T)** Quantification of the central time (**R**) and total distance (**S**) and the example traces (**T**) in the OFT from the mCherry and eNpHR3.0-mCherry group (unpaired t-test, *n* = 7/group). **(U-W)** Quantification of the time (**U**) and entries (**V**) in open arms and the example traces (**W**) in the EPM from the mCherry and eNpHR3.0-mCherry group (unpaired t-test, *n* = 7/group). **(X**,** Y)** Immobility time in TST (**X**) and FST (**Y**) from the mCherry and eNpHR3.0-mCherry group (unpaired t-test, *n* = 7/group). Data were presented as the mean ± SEM. *P < 0.05, **P < 0.01, ***P < 0.001, ns = no significant

To determine the functional role of the mOFC^CaMKIIα^-MD pathway after nerve injury, we selectively inhibited mOFC^CaMKIIα^ neurons projecting to the MD using the chemogenetic approach. We injected AAV-CaMKII-Cre into mOFC and rAAV2-retro-hSyn-DIO-hM4D(Gi)-mCherry into MD. Successful expression of hM4D(Gi)-mCherry was observed in mOFC^CaMKIIα^ projecting to the MD (Fig. 5A). The timeline of viral injection and behavioral testing is shown in Fig. 5B. CNO administration significantly ameliorated CCI-induced mechanical and thermal hypersensitivity in mice expressing hM4D(Gi)-mCherry compared with control group(Fig. 5C-E). However, no significant differences in locomotor activity or anxiodepressive-like behaviors were observed between the two groups following CNO administration, as assessed by the OFT, EPM, TST, and FST (Fig. 5F-M). To determine whether activation of the mOFC^CaMKIIα^-MD projection induces pain-related behaviors in naïve mice, we injected AAV-CaMKII-Cre into the mOFC and rAAV2-retro-hSyn-DIO-hM3D(Gq)-EGFP into the MD (Fig. S5A). Successful expression of hM3D(Gq)-EGFP was observed in mOFC (Fig. S5B). Chemogenetic activation of the mOFC^CaMKIIα^-MD pathway produced significant increases in pain sensitivity in naïve mice, as evidenced by increased paw withdrawal frequency and reduced paw withdrawal latencies, whereas anxiodepressive-like behaviors remained unchanged across all behavioral paradigms (Fig. S5D-L). Consistent with prior studies, chemogenetic activation of the MD in mice induces hyperalgesia [37], suggesting that the MD plays a critical role in nociceptive modulation.

We next used optogenetics to further verify the modulation of the mOFC^CaMKIIα^-MD pathway in pain behaviors after CCI. AAV2/9-CaMKII-eNpHR3.0-mCherry was injected into the mOFC to selectively express the inhibitory opsin eNpHR3.0 in CaMKIIα^+^ neurons, and an optical fiber was implanted above the MD to inhibit the axon terminals of mOFC-MD projections. Robust mCherry fluorescence was detected in the axonal terminals of the MD (Fig. 5N). Optical inhibition of mOFC^CaMKIIα^ terminals within MD significantly alleviated CCI-induced mechanical and thermal hypersensitivity (Fig. 5O-Q), consistent with the chemogenetic results. Optogenetic inhibition did not affect locomotor or anxiodepressive-like behaviors, as assessed by the OFT, EPM, TST, and FST (Fig. 5R-Y). As a complementary approach, we injected AAV2/9-CaMKII-ChR2-mCherry into mOFC and implanted an optical fiber above MD. Optogenetic activation of the mOFC^CaMKIIα^-MD pathway produced behavioral effects that partially overlapped with those observed following chemogenetic activation (Fig. S5M-X). Notably, optogenetic activation resulted in a significant reduction in both open-arm time and entries in the elevated plus maze (Fig. S5U, V), which was not observed following chemogenetic activation. These findings provide convergent evidence that suppression of glutamatergic output from mOFC to MD attenuates pain hypersensitivity after nerve injury, without affecting anxiodepressive-like behaviors.

### Selective inhibition of mOFC^CaMKIIα^-BLA pathway leads to amelioration of CCI-induced anxiodepressive-like behaviors

**Fig. 6:**
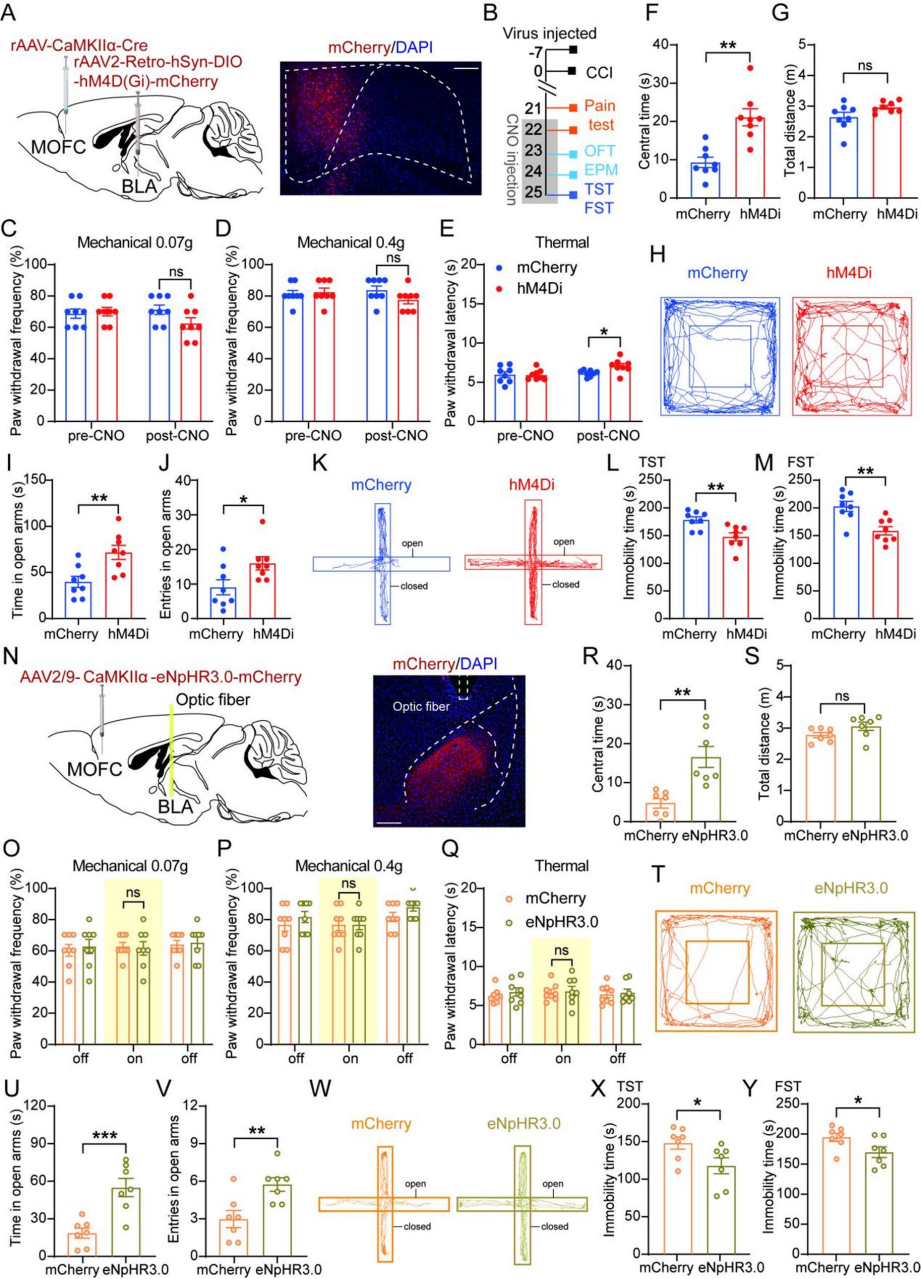
Chemogenetic and optogenetic inhibition of mOFC^CaMKIIα^-BLA projections alleviates CCI-induced anxiodepressive-like behaviors. (**A**) Schematic of experimental design (left) and the expression of hM4D(Gi)-mCherry in the mOFC (right, AP = + 2.45 mm). Scale bar: 100 μm. (**B**) Viral injection and behavioral tests timeline. (**C**-**E**) Effect of chemogenetic inhibition of mOFC^CaMKIIα^ - BLA pathway neurons on pain behavior in 0.07 g (**C**), 0.4 g (**D**) von Frey and Hargreaves tests (**E**) (two-way ANOVA with Sidak post hoc test, *n* = 8/group). (**F**-**H**) Quantification of the central time (**F**) and total distance (**G**) and the example traces (**H**) in the OFT from the mCherry and hM4D(Gi)-mCherry group (unpaired t-test, *n* = 8/group). (**I**-**K**) Quantification of the time (**I**, unpaired t-test) and entries (**J**, Mann-Whitney test) in open arms and the example traces (**K**) in the EPM from the mCherry and hM4D(Gi)-mCherry group (*n* = 8/group). (**L**, **M**) Immobility time in TST (**L**) and FST (**M**) from the mCherry and hM4D(Gi)-mCherry group (unpaired t-test, *n* = 8/group). (**N**) Schematic of experimental design (left) and the axon terminals expression of eNpHR3.0-mCherry and optic fiber implantation in the BLA (right, AP = − 1.31 mm). Scale bar: 100 μm. (**O**-**Q**) Effect of optogenetic inhibition of mOFC^CaMKIIα^-BLA pathway neurons on pain behavior in 0.07 g (**O**), 0.4 g (**P**) von Frey and Hargreaves tests (**Q**) (two-way ANOVA with Sidak post hoc test, *n* = 8/group). (**R**-**T**) Quantification of the central time (**R**) and total distance (**S**) and the example traces (**T**) in the OFT from the mCherry and eNpHR3.0-mCherry group (unpaired t-test, *n* = 7/group). (**U**-**W**) Quantification of the time (**U**, unpaired t-test) and entries (**V**, Mann-Whitney test) in open arms and the example traces (**W**) in the EPM from the mCherry and eNpHR3.0-mCherry group (*n* = 7/group). (**X**, **Y**) Immobility time in TST (**X**) and FST (**Y**) from the mCherry and eNpHR3.0-mCherry group (unpaired t-test, *n* = 7/group). Data were presented as the mean ± SEM. *P < 0.05, **P < 0.01, ***P < 0.001, ns = no significant

We next focused on the functional role of the mOFC^CaMKIIα^-BLA pathway after nerve injury. To selectively inhibit mOFC^CaMKIIα^ neurons projecting to the BLA using a chemogenetic approach, we injected AAV-CaMKII-Cre into mOFC, and rAAV2-retro-hSyn-DIO-hM4D(Gi)-mCherry into BLA. Successful expression of hM4D(Gi)-mCherry was observed in mOFC^CaMKIIα^ projecting to the BLA (Fig. 6A). CNO administration did not alter mechanical pain hypersensitivity and only produced a mild attenuation of thermal hyperalgesia in CCI mice (Fig. 6C-E). In contrast, inhibition of this pathway significantly ameliorated CCI-induced anxiodepressive-like behaviors, as evidenced by elevated center time in the OFT, increased entries and time spent in the open arms of the EPM, and reduced immobility time in the TST and FST (Fig. 6F-M). Importantly, total travel distance in the OFT was not affected by CNO administration, suggesting that the observed behavioral changes were not due to alterations in motor function. To determine whether activation of the mOFC^CaMKIIα^-BLA projection induces anxiodepressive-like behaviors in naïve mice, we injected AAV-CaMKII-Cre into the mOFC and rAAV2-retro-hSyn-DIO-hM3D(Gq)-EGFP into the BLA (Fig. S6A). Successful expression of hM3D(Gq)-EGFP was observed in mOFC (Fig. S6B). Although mechanical pain sensitivity remained unchanged, a slight reduction in thermal withdrawal latency was observed, which might be attributed to collateral projections of mOFC^CaMKIIα^ neurons (Fig. S6D-F). Notably, chemogenetic activation of the mOFC^CaMKIIα^-BLA pathway induced anxiodepressive-like behaviors in OFT, EPM, TST, and FST tests in naïve mice, suggesting that this pathway mainly modulates the affective component of pain (Fig. S6G-L).

Optogenetic manipulation was used to further verify the role of the mOFC^CaMKIIα^-BLA pathway in anxiodepressive-like behaviors following CCI. AAV2/9-CaMKII-eNpHR3.0-mCherry was injected into the mOFC, and an optical fiber was implanted above the BLA to inhibit the axon terminals of mOFC^CaMKIIα^-BLA projections. Robust mCherry fluorescence was detected in the axonal terminals of the BLA (Fig. 6N). Optical inhibition of mOFC^CaMKIIα^ terminals within BLA did not alter CCI-induced mechanical and thermal hypersensitivity (Fig. 6O-Q), consistent with the chemogenetic results. Similar to the chemogenetic results, optogenetic inhibition ameliorated anxiodepressive-like behaviors after nerve injury, as assessed by the OFT, EPM, TST, and FST (Fig. 6R-Y). As a complementary approach, we injected AAV2/9-CaMKII-ChR2-mCherry into mOFC and implanted an optical fiber above BLA. Optogenetic activation of mOFC^CaMKIIα^-BLA pathway produced results consistent with those obtained from chemogenetic activation (Fig. S6M-X). These results indicate that the mOFC^CaMKIIα^-BLA pathway primarily modulates the affective dimension rather than the sensory dimension of neuropathic pain.

## Discussion

In the present study, we provide insight into the enhanced activity of mOFC glutamatergic neurons is essential for nerve injury-induced pain hypersensitivity and anxiodepressive behaviour. Our results showed that inhibition of excitatory neurons in the mOFC improved the somatosensory and affective components of neuropathic pain. Furthermore, through viral tracing, chemogenetic, and optogenetic approaches, we determined that the mOFC^CaMKIIα^-MD pathway modulates pain hypersensitivity, while the mOFC^CaMKIIα^-BLA pathway is mainly involved in modulating the anxiodepressive behaviour.

Consistent with the previous study [3], we observed notable pain hypersensitivity and anxiodepressive-like phenotypes in CCI model (Fig. 1A-K). Converging evidence suggests that neuropathic pain and affective disorders share common neural circuits and molecular mechanisms [16, 38]. For instance, increased proinflammatory signaling enhances nociceptor sensitization and depressive symptoms [39, 40]. Circuit-level studies further support this interconnection: activation of serotonergic projections from the dorsal raphe nucleus to the central nucleus of the amygdala attenuated SNI-induced pain hypersensitivity and depression-like behaviors [41]. In addition, recent work has demonstrated that inhibition of glutamatergic projections from the VTA^Glu^ to the NAc alleviates both pain hypersensitivity and pain-related anxiety following nerve injury [42]. Chronic pain is associated with widespread changes in neural activity and connectivity across multiple brain regions. These regions include corticolimbic circuits, prefrontal cortex, anterior cingulate cortex, and thalamic areas, which are involved in sensory, affective, and cognitive processing [9, 43, 44]. We observed that mOFC glutamatergic neurons were activated in neuropathic pain mice displaying anxiodepressive-like phenotypes, as shown by electrophysiological recordings and fiber photometry in freely moving mice (Fig. 1L-Z). Functionally, inhibition of these neurons effectively alleviated both pain hypersensitivity and anxiodepressive-like behaviors following nerve injury. Notably, suppressing mOFC glutamatergic activity in uninjured mice had no detectable effect on baseline nociceptive thresholds or emotional behaviors, indicating that these neurons are not required for the maintenance of sensory or affective states under physiological conditions. Moreover, chemogenetic activation of mOFC glutamatergic neurons in CCI mice did not further exacerbate pain hypersensitivity or affective symptoms, suggesting the presence of a ceiling effect once these neurons are engaged under neuropathic pain conditions. Taken together, these findings indicate that mOFC glutamatergic neurons are selectively recruited following nerve injury and operate in an “all-or-none” mode to drive both sensory and affective disturbances.

The OFC receives multi-sensory inputs and sends reciprocal projections to several subcortical structures, positioning it as a key hub for emotional regulation, decision-making, and behavioral flexibility [45, 46]. To further investigate whether mOFC is involved in regulating the sensory and affective components of neuropathic pain through subcortical projections, we used cre-dependent viral tracing to map the downstream targets of mOFC^CaMKIIα^ neurons. Consistent with previous tracer studies [47, 48], our immunohistochemistry results showed that glutamatergic neurons in mOFC projected densely to both the MD and BLA (Fig. 4A-D). We then used specific retrograde labeling to determine projection patterns of mOFC neurons to the MD and BLA. This result revealed two non-overlapping mOFC^CaMKIIα^ neurons projecting to the MD and BLA, respectively (Fig. 4E-I), indicating a projection-defined organizational segregation within the mOFC. It has long been hypothesized that central neurons are organized into functionally distinct ensembles or engrams, such that a select population of neurons encodes a specific behavioral output [49, 50]. This hypothesis emphasizes the functional distinction between neuronal ensembles [51]. Given these anatomically distinct projection patterns, we want to explore whether the different projection patterns of mOFC glutamate neurons to MD and BLA mediate the modulation of neuropathic pain and anxiodepressive-like behaviors separately.

There is evidence that MD constitutes an integral component of the medial nociceptive transmission system [52]. The MD can be activated by nociceptive stimuli and chemogenetic activation of the MD or the ACC-MD pathway induced hyperalgesia alongside aversion and anxiety-like behaviors [37]. In addition, inhibition of prelimbic (PL) cortex projections to the MD has been reported to enhance capsaicin-induced pain behavior [11], highlighting the role of prefrontal cortex-thalamic circuits in modulating nociceptive processing. Our findings indicate that the mOFC^CaMKIIα^-MD projection selectively contributes to the sensory dimension of neuropathic pain. Specifically, modulation of this pathway influences mechanical and thermal hypersensitivity without altering anxiety- or depression-like behaviors, suggesting a functional dissociation between mOFC output pathways in regulating sensory and affective components of chronic pain.

The basolateral amygdala (BLA) is a central hub for emotional processing and social cognition and plays a critical role in integrating affective and sensory information [53, 54]. Several imaging studies have reported that abnormal connectivity between the mOFC and amygdala was observed in patients with a history of early-life social adversity [55, 56]. Furthermore, recent study has demonstrated that optogenetic and chemogenetic manipulation of the orbitofrontal cortex-amygdala circuit directly regulates emotional and social behaviors in rodents [10]. Interestingly, optogenetic inhibition of the BLA to anterior cingulate cortex projection selectively inhibited the chronic pain induced depression-like behaviors, yet the pain threshold was intact [57]. In the present study, we extend these observations by demonstrating that the mOFC^CaMKIIα^-BLA projection contributes predominantly to the affective dimension of neuropathic pain. Selective inhibition of this pathway in CCI mice robustly alleviated anxiety- and depression-like behaviors (Fig. 6), indicating that enhanced mOFC-BLA signaling is required for the maintenance of pain-associated affective disturbances. Notably, although manipulation of this pathway had minimal effects on mechanical hypersensitivity, inhibition of mOFC-BLA neurons modestly reduced thermal hyperalgesia (Fig. 6E), while activation in naïve mice increased thermal pain sensitivity (Figure S6F). These findings suggest that, beyond its primary role in affective regulation, the mOFC-BLA pathway may indirectly influence thermal nociceptive processing. One possible explanation is that mOFC glutamatergic neurons give rise to collateral projections that engage other downstream circuits involved in the thermal pain modulation [58]. Alternatively, growing evidence indicates that amygdala-centered circuits interact with prefrontal and midbrain structures to shape nociceptive responses in pain state [59, 60]. In particular, BLA-PFC-PAG pathways has been identified that contributes to mechanical and thermal hypersensitivity after nerve injury [61]. Activation of this pathway strengthens BLA inputs onto inhibitory neurons in PFC, altering descending modulation from PFC to PAG and ultimately affecting spinal nociceptive processing. These findings support the notion that affective and sensory dimensions of pain are not strictly segregated, but instead exhibit functional interdependence at the level of neural circuits. Taken together, our results indicate that the mOFC^CaMKIIα^-BLA circuit not only critically regulates the affective dimension of neuropathic pain, but also can modulate specific sensory components, such as thermal nociception.

In addition to MD and BLA projections, the mOFC also sends excitatory outputs to other regions involved in pain and affective processing, including the ACC, NAc, agranular insular cortex (AI), centrolateral thalamus (CL), and ventromedial thalamus (VM). The ACC is well known to play a critical role in the affective and motivational aspects of chronic pain. Both human imaging and animal studies have shown altered activity, synaptic plasticity, and changes in prefrontal-ACC connectivity in neuropathic pain models [62, 63]. Projections from mOFC to ACC may thus influence the emotional evaluation of pain and decision-making related to avoidance behavior. The NAc, while traditionally associated with reward processing, also contributes to nociceptive processing [64]. Recent studies have shown that projections from prefrontal regions to the NAc modulate both sensory and affective components of pain, with optogenetic inhibition of PFC-NAc inputs increasing nociceptive sensitivity and aversive behaviors in chronic pain models [65]. Therefore, mOFC-NAc inputs may be involved in regulating both sensory and affective aspects of pain in rodents. Additionally, the claustrum and anterior insular cortex (AI) are anatomically adjacent and highly interconnected, forming a hub for sensory and affective information. The anterior insular cortex is necessary for empathetic pain perception [66], while the claustrum is involved in processing pain-related sensory signals [67]. Thus, mOFC projections to the claustrum-AI complex could enable the orbitofrontal cortex to modulate the integration of sensory inputs with emotional evaluation, shaping the overall pain experience. The ventromedial (VM) thalamus has been recognized as a key node for endogenous pain modulation, particularly in regulating thermal nociception [52]. Unlike MD, which facilitates mechanical hypersensitivity, VM can engage descending inhibitory pathways to reduce thermal pain [68]. These findings suggest that mOFC projections to VM may modulate thermal pain sensitivity through top-down control within thalamocortical circuits. Collectively, these findings suggest that the mOFC exerts control over both sensory and affective dimensions of neuropathic pain through distinct output pathways, coordinating thalamic, limbic, and cortical circuits to shape the overall pain experience.

Pain perception and modulation emerge from the coordinated activity of distributed cortical-subcortical circuits, commonly conceptualized as the pain matrix. Within this framework, prefrontal regions operate as higher-order modulatory hubs that exert top-down control over thalamic, limbic, and insular circuits [43]. Alterations in one prefrontal node are therefore likely to influence activity across the broader network. Brain regions adjacent to the mOFC, such as the PL and rostral anterior cingulate cortex (rACC), have also been shown to regulate pain sensitivity and anxiety under chronic pain conditions [44, 69]. Although direct correlations between mOFC activity and these regions were not examined in the present study, their close anatomical proximity and functional overlap suggest that neuropathic pain may engage coordinated activity changes across prefrontal and cingulate cortices. Accordingly, our findings support the view that the mOFC acts as an integral cortical component of the pain matrix, coordinating sensory and affective dimensions of chronic pain through its distinct output pathways, rather than functioning independently of nearby prefrontal or cingulate regions.

Supported by these findings, we demonstrated that divergent mOFC neural circuits independently mediate the sensory and affective dimensions of neuropathic pain. Although this study delineates projection-specific mOFC circuits involved in neuropathic pain, several limitations should be acknowledged. The downstream neuronal subtypes within the MD and BLA that receive mOFC inputs were not identified, leaving the postsynaptic mechanisms unresolved. Other mOFC-connected regions, such as the ACC, NAc, or VM, may also participate in pain and affective modulation but were not verified here. Future work should combine cell-type-specific tracing and functional mapping to clarify these issues.

In conclusion, our study highlights the critical involvement of mOFC glutamatergic neurons in the comorbidity of chronic pain and affective disturbances. These neurons are activated in neuropathic pain mice accompanied by anxiodepressive-like phenotypes. Chemogenetic and optogenetic inhibition of mOFC glutamatergic neurons attenuates both pain hypersensitivity and anxiodepressive-like behaviors after nerve injury. Distinct output pathways from the mOFC separately control the sensory and affective manifestations of neuropathic pain. The glutamatergic outputs from mOFC to BLA contributed to the regulation of anxiodepression-like behaviors. In contrast, the glutamatergic outputs from mOFC to MD mediated pain hypersensitivity. These findings provide a new perspective for treating chronic pain-associated emotional comorbidities.

## Materials and methods

### Animals

Adult male C57BL/6J, Ai14 (Rosa26-LSL Tdtomato), and *CaMKIIα-Cre* mice (8–10 weeks old) were used for all experiments. C57BL/6 mice were purchased from the Experimental Animal Center of Zhengzhou University. *CaMKII-Cre* and *Ai-14* mice were purchased from the Shanghai Model Organisms Center. To identify CaMKIIα + neurons, *CaMKII-Cre* male mice were crossed with Ai14 tdTomato female mice to obtain *CaMKII-Cre*,* Ai14* mice with tdTomato fluorescence in Cre-expressing cells (CaMKII-Cre; tdTomato). Mice were housed under standard laboratory conditions (25 ± 1 °C) with ad libitum access to food and water and maintained on a 12-hour light/dark cycle. All procedures were approved by the Animal Center of Henan Province and adhered to national guidelines for laboratory animal welfare.

### Chronic constriction injury (CCI) surgery

The CCI surgery was carried out as described previously while with minor modifications [70]. Mice were anesthetized with inhaled isoflurane in 100% oxygen (induced at 4–5%; maintained at 2–3%). In the CCI group, the left sciatic nerve was exposed, and three 6 − 0 chromic catgut ligatures were loosely tied around the nerve with 1.5 mm spacing. The sham group underwent an identical procedure, but without the ligatures. The animals were randomly assigned to either the CCI or the Sham group.

### Behavioral testing

#### Paw withdrawal frequency (PWF)

To assess mechanical hypersensitivity, paw withdrawal frequency (PWF) in response to calibrated mechanical stimuli was measured using two calibrated von Frey filaments (0.07 and 0.4 g), as described previously [71]. Mice were individually placed on an elevated wire mesh platform within transparent chambers and acclimated for at least 45 min. The filament was applied perpendicularly to the central plantar surface of the hind paw for ~ 1–2 s, repeated 10 times with at least 10-second intervals between each stimulus. The number of paw withdrawal responses was recorded and expressed as a percentage.

#### Paw withdrawal latency (PWL)

The paw withdrawal latencies (PWL) to heat stimuli in mice were measured with the Hargreaves apparatus (Model 390; IITC Life Science Inc, Woodland Hills, CA) as described previously [72]. The mice were acclimated on a glass plate inside a plastic box for 45 min before the test. Subsequently, a radiant heat was applied by aiming a beam of light through a hole in the lightbox through the glass plate to the middle of the plantar surface of each hind paw. When the animal lifted its foot, the light beam was turned off. The length of time between the start of the light beam and the foot lift was defined as the paw withdrawal latency. Each trial was repeated 3 times at 10-minute intervals each. Behavioral testing was conducted at baseline and on days 1, 3, 5, and 7 post-injection to assess the development and persistence of hyperalgesia.

#### Open field test (OFT)

For OFT, mice were acclimated to the behavioral testing room for at least 1 h prior to the experiment to minimize stress-related variability. When it began, mice were placed in the center of an open field apparatus, which was a yellow Plexiglas box (40 cm × 40 cm × 50 cm). Their behavior was recorded for 5 min using a computer-connected camera and measured and analyzed using the Smart v3.0 video tracking system (Harvard Apparatus; Panlab, Barcelona, Spain). After each test, the open-field arena was cleaned with 75% ethanol. The total distance traveled and time spent in the central zone were analyzed as indices of locomotor activity and anxiety-like behavior, respectively.

#### Elevated plus maze test (EPM)

For the EPM test, mice were acclimated to the behavioral testing room for at least 1 h prior to the experiment to minimize stress-related variability. The elevated plus maze was positioned 60 cm above the floor and consisted of a central platform (5 cm × 5 cm), two open arms (30 cm × 6 cm × 15 cm), and two closed arms (30 cm × 6 cm × 15 cm). Mice were placed on the central platform facing an open arm and allowed to explore freely for 5 min in a dimly lit room. The total distance traveled, the time spent in the open arms and entries into the open arms were recorded using the Smart v3.0 software (Harvard Apparatus; Panlab). The arena was cleaned with 75% ethanol between trials to eliminate olfactory cues. Time spent in the open arms and entries into the open arms were used as an index of anxiety-like behavior.

#### Tail suspension test (TST)

For the tail suspension test, mice were suspended by their tails (proximately 2 cm from the tip, proximately 30 cm above the ground) using a piece of medical tape. During the 6-min testing period, the animals were videotaped and total immobility (reflecting behavioral despair; in which the mice hung without any limb movement) for 4 min. The duration of immobility during the final 4 min of the test was quantified.

#### Forced swimming test (FST)

For the forced swimming test (FST), mice were placed into Plexiglass cylinders (15 cm diameter × 30 cm height) filled with 18 cm water (temperature: 25 ± 1 °C;) for 6 min. The water level was set to prevent the mice from touching the bottom with their limbs or tails. The test is under normal illumination with a digital video camera recording from the side. Only the immobility time (which was defined as the time when mice floated passively without any movements other than the necessary ones to keep their bodies balanced) during the 2nd -6th minutes of the test was scored by a trained observer.

### Stereotaxic surgery and virus injection

The mice were anesthetized using isoflurane inhalation (induction at 2%-4%; maintenance at 1%-2%) and positioned on a stereotaxic apparatus (RWD Life Science, Shenzhen, China). Erythromycin eye ointment was applied to prevent corneal drying, and a heating pad was used to maintain a body temperature of 37 °C. Viruses were then injected into the mOFC, BLA or MD (mOFC: coordinates: AP = + 2.35, ML = +/−0.25, DV = − 2.6; BLA: AP = -1.5, ML = +/−3.00, DV = − 4.70; MD: AP = -1.20, ML = +/−0.35, DV = − 3.25) [51] using a modified 1 µl Hamilton microsyringe connected to a microsyringe. The injection was performed at a controlled rate of 10–15 nL/minute. After the injection, the needle remained in place for an additional 10 min before retraction to prevent backflow. Experiments were performed at least 3 weeks after the vector injection.

For recording calcium imaging data, the rAAV2/9-CaMKIIa-GCaMP6s-WPRE-hGH polyA was injected into the mOFC of C57 mice. After 3 weeks, an optical fiber was implanted towards the mOFC and then secured to the skull using dental cement.

For selective chemogenetic activation or inhibition of the mOFC glutamatergic neurons, the rAAV2/9-CaMKIIa-hM4D(Gi)-mCherry-WPRE or rAAV2/9-CaMKIIa-hM3D(Gq)-EGFP-WPRE was injected into the mOFC of C57 mice.

For selective Optogenetic activation or inhibition of the mOFC glutamatergic neurons, the rAAV2/9-CaMKIIa-eNpHR3.0-mCherry-WPRE or rAAV2/9-CaMKIIa-ChR2-mCherry-WPRE was injected into the mOFC of C57 mice.

For anterograde tracing of the mOFC, rAAV2/9-hSyn-DIO-mGFP-T2A-Synaptophysin-mRuby- WPRE was injected into the mOFC of *CaMKII-Cre* mice. Three weeks later, the mice were killed, and whole-brain sectioning was performed to locate the downstream nuclei of the mOFC.

For retrograde tracing from the BLA and MD to mOFC, the AAV2/2Retro-hsyn-EGFP and AAV2/2Retro-hsyn-mCherry were injected separately into the BLA and MD of C57 mice; the AAV2/2Retro-hEf1α-DIO-EGFP and AAV2/2Retro-hEf1α-DIO-mCherry were injected separately into the BLA and MD of *CaMKII-Cre* mice.

For optogenetic activation or inhibition of the mOFC^CaMKIIa^-BLA/MD circuits, the rAAV2/9-CaMKIIa-ChR2-mCherry or rAAV2/9-CaMKIIa-eNpHR3.0-mCherry was injected into the mOFC of C57 mice. After 3 weeks, an optical fiber was implanted towards the BLA/MD, and then secured to the skull using dental cement.

For chemogenetic activation or inhibition of the mOFC^CaMKIIa^-BLA/MD circuits, the rAAV2/9-CaMKIIa-Cre-WPRE was injected into the mOFC, and the AAV2/2Retro-hsyn- DIO-hM3D(Gq)-mCherry or AAV2/2Retro-hsyn-DIO-hM4D(Gi)-mCherry was injected into the BLA/MD of C57 mice.

### Chemogenetic and optogenetic activation

For chemogenetic manipulation, clozapine-N-oxide (CNO, Sigma-Aldrich) was dissolved in sterile saline and administered via intraperitoneal (i.p.) injection at a dose of 2 mg/kg to selectively activate excitatory (hM3Dq) or inhibitory (hM4Di) DREADD receptors. Injections were typically performed 30 min prior to behavioral testing to allow sufficient time for CNO to exert its effects. Control animals expressing mCherry or EGFP also received CNO injections at the same dose (2 mg/kg) as the experimental groups.

For optogenetic stimulation, mice received stereotaxic injections of AAV. Following viral injection, optical fiber cannulas (200 μm core diameter) were implanted above mOFC using the same stereotaxic coordinates. The fibers were secured to the skull using dental cement to ensure long-term stability. Optogenetic stimulation was performed using 473 nm (blue) and 589 nm (yellow) lasers coupled to implanted optical fibers. For blue-light activation of ChR2, light pulses were delivered at 10 ms pulse width and 20 Hz, with the output power at the fiber tip set to approximately 6–8 mW [73]. For yellow-light inhibition of eNpHR3.0, continuous illumination at ~ 8 mW at the fiber tip was applied throughout the behavioral testing. Laser parameters were controlled using a pre-programmed stimulation system to ensure precise timing and consistency across animals. Control animals received injections of control AAVs expressing mCherry or EGFP and underwent the same surgical and optical stimulation procedures.

All procedures were conducted under aseptic conditions, and animals were allowed to recover for at least 3 weeks post-surgery to ensure adequate expression of DREADDs or opsins before experimental manipulation. After completion of behavioral experiments, mice were perfused transcardially and brains were sectioned coronally. The positions of optic fiber tips were verified by identifying the fiber track in combination with viral fluorescence and anatomical landmarks according to a mouse brain atlas.

### Immunofluorescent histochemical staining

The experimental procedure was performed as previously described [74]. Animals were deeply anesthetized with overdose of pentobarbital sodium (100 mg/kg, i.p.), and then perfused transcardially with 50 ml of 0.01 M phosphate-buffered saline (0.9%Nacl, pH 7.4) before 100 ml of paraformaldehyde (PFA). The brain tissue was promptly excised and subjected to post-fixation. The brains was removed and immersed in 30% sucrose dissolved in 0.1 M PB after 4% PFA until they sank to the bottom of the container. All brains were cut into 35 μm transverse sections and collected sequentially in a six-well culture plate on a freezing microtome (CM1950; Leica, Heidelberg, Germany), and stored in PBS at 4 °C until further processing. Sections containing injection sites or target nuclei were observed under an epifluorescence microscope (BX-60; Olympus, Tokyo, Japan) to confirm the accuracy of injections.

### Whole-cell patch-clamp recording

Brain slices containing the mOFC of CaMKII-Cre, Ai14 mice were prepared following procedures similar to those described previously [75]. The 300 μm-thick brain slices were prepared using a vibratome (VT 1200s; Leica) in ice-cold, oxygenated cutting buffer (95% O_2_/5% CO_2_), containing 213 mM sucrose、3 mM KCl、1.25 mM NaH_2_PO_4_、0.5 mM CaCl_2_、4.5 mM MgSO_4_、26 mM NaHCO_3_、10 mM D-Glucose、1.3 mM Sodium ascorbate 、0.6 mM Sodium pyrurate 、pH 7.4. The slices were then transferred to normal sodium-based artificial cerebrospinal fluid, which consisted of 125 mM NaCl、3 mM KCl、1.25 mM NaH_2_PO_4_、2 mM CaCl_2_、1 mM MgSO_4_、26 mM NaHCO_3_、10 mM D-Glucose、1.3 mM Sodium ascorbate 、0.6 mM Sodium pyrurate 、pH 7.4. Recordings of mOFC glutamatergic neurons, identified as bright cells under infrared differential interference contrast microscopy, were performed in current-clamp modes using a Multiclamp 700B amplifier (Axon Instruments, Foster City, CA, United States). The data were analyzed using pCLAMP v.11.3 software. Whole-cell patch-clamp was achieved after forming a giga-seal (> 1 GΩ) between the pipette tip (4–6 MΩ) and the neuronal membrane. Series resistance was continuously monitored, and recordings were discarded if resistance changed by more than 15%. During recordings, cells were perfused with a bath solution at room temperature (22–25 °C). The current clamp (holding current I = 0 mA) was used to measure resting membrane potential and to record neuronal responses to injected currents. For action potential number testing, the protocol was configured as stepwise 500 milliseconds current injections ranging from 0 to 350 pA in 25 pA increments.

### Data analysis

All data were expressed as mean ± SEM and were analyzed using GraphPad Prism 9.5 software (GraphPad Software, Inc., La Jolla, CA, United States). For comparisons between two groups, an unpaired Student’s *t*-test was employed. For comparisons involving more than two groups, one-way or two-way analysis of variance followed by the Sidak post hoc test was used. Statistical significance was set at *P* < 0.05.

## Supplementary Information

Below is the link to the electronic supplementary material.

Supplementary Material 1

Supplementary Material 2

## Data Availability

No datasets were generated or analysed during the current study.
