# Supplementary material for: The medial orbitofrontal cortex mediates neuropathic pain and anxiodepressive-like behaviors via two distinct pathways
Source: J Headache Pain. 2026 Mar 18;27(1):119. doi: 10.1186/s10194-026-02335-w (PMC13112918; doi:10.1186/s10194-026-02335-w)
Supplement: Supplementary file 1 — Supplementary Material 1 [file 10194_2026_2335_MOESM1_ESM.docx]

**
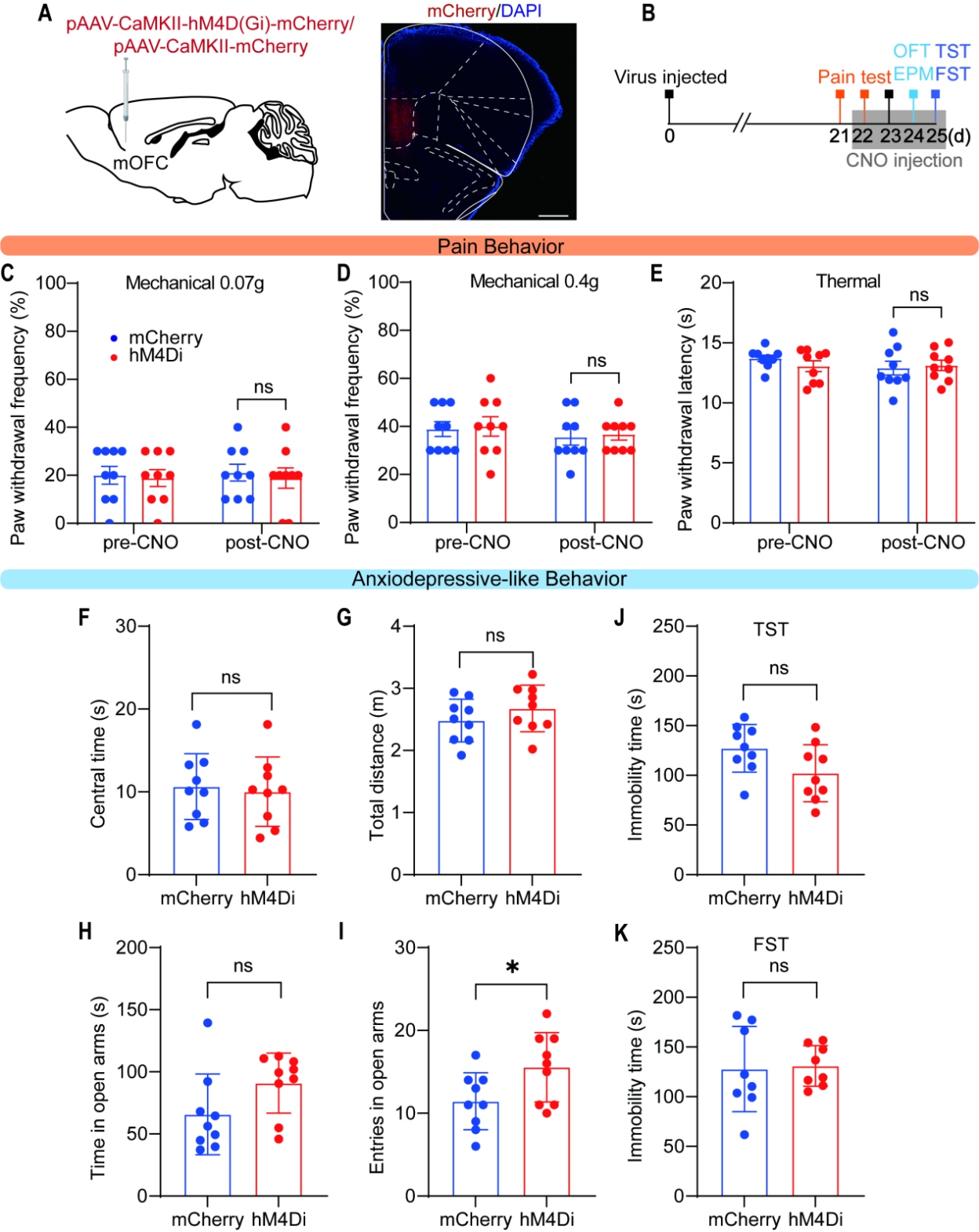
**

**Figure S1. Inhibition of mOFC glutamatergic neurons in normal mice does not alter pain or anxiodepressive-like behaviors. (A)** Schematic diagram of stereotaxic delivery of AAV-CaMKII- hM4D(Gi)-mCherry (left) in Sham mice and representative images indicated the injection site was mOFC (right, AP = + 2.30 mm). Scale bar: 100 μm **(B)** Behavioral tests timeline. **(C-E)** Effect of chemogenetic inhibition of mOFC glutamatergic neurons on pain behavior in 0.07g (C), 0.4g (D) von Frey and Hargreaves tests (E) (two-way ANOVA with Sidak post hoc test, n=9/group). **(F-K)** Effect of chemogenetic inhibition of mOFC glutamatergic neurons on anxiodepressive-like behavior in Central time (F, unpaired t-test), Total distance (G, unpaired t-test), Time (H, Mann-Whitney test) and entries in open arms (I, unpaired t-test), TST (J), FST (K, unpaired t-test), (n=9/group). Data were presented as the mean ± SEM. *P < 0.05, **P <0.01, ***P < 0.001, ns = no significant.

**
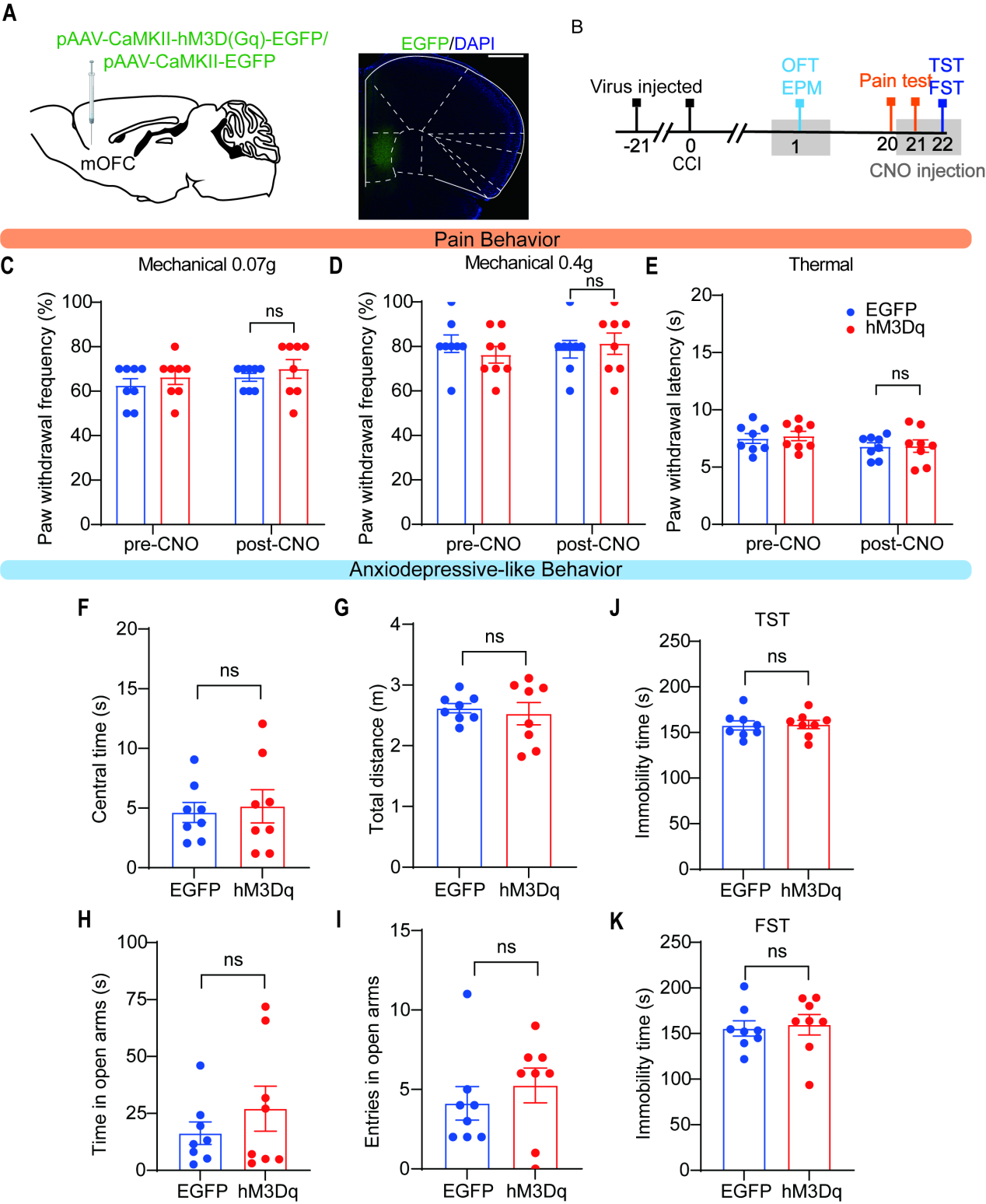
**

**Figure S2. Activation of mOFC glutamatergic neurons in CCI mice does not exacerbate pain or anxiodepressive-like behaviors. (A)** Schematic diagram of stereotaxic delivery of AAV-CaMKII-hM3D(Gq)-EGFP (left) and representative images indicated the injection site was mOFC (right, AP = + 2.30 mm). Scale bar: 100 μm **(B)** Behavioral tests timeline **(C-E)** Effect of chemogenetic activation of mOFC glutamatergic neurons on pain behavior in 0.07g (C), 0.4g (D) von Frey and Hargreaves tests (E) (two-way ANOVA with Sidak post hoc test, n=8 mice/group). **(F-K)** Effect of chemogenetic activation of mOFC glutamatergic neurons on anxiodepressive-like behavior in Central time (F, unpaired t-test), Total distance (G, unpaired t-test), Time in open arms (H, Mann-Whitney test), Entries in open arms (I, Mann-Whitney test), TST (J, unpaired t-test), FST (K, unpaired t-test), (n=8 mice/group). Data were presented as the mean ± SEM. *P < 0.05, **P <0.01, ***P < 0.001, ns = no significant.

**
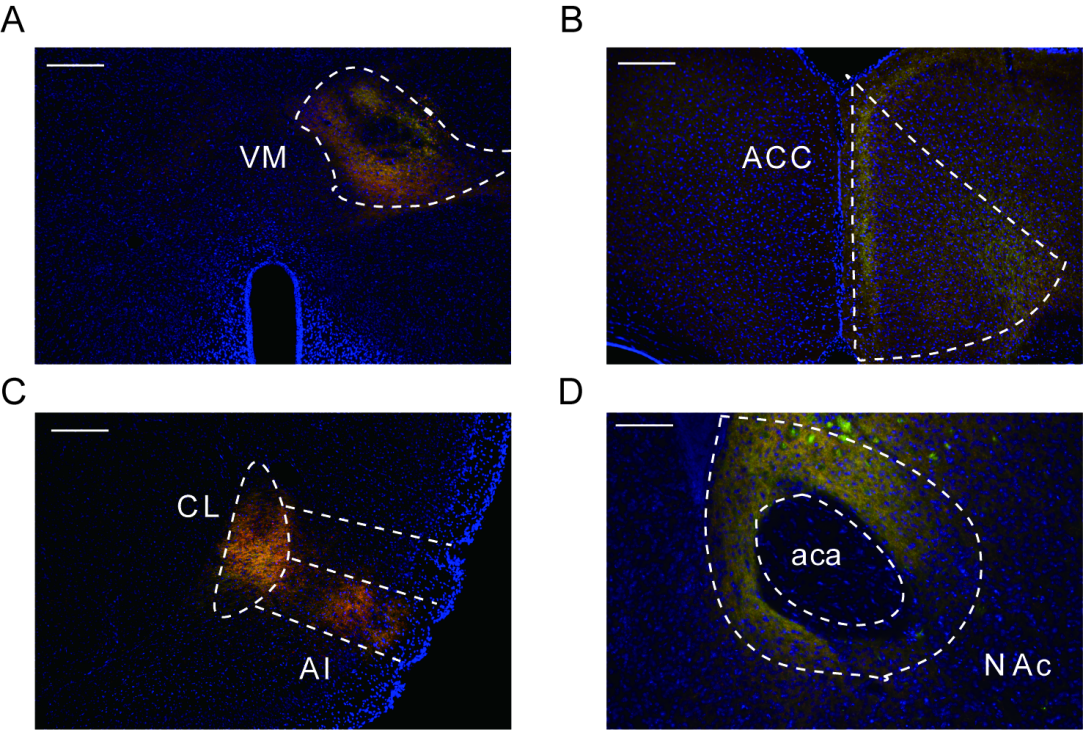
**

**Figure S3** **Tracing result of AAV-DIO-mGFP-2A-Synaptophysin-mRuby in the mOFC of *CaMKII-Cre* mice. (A-D)** Representative images showing that the mOFC CaMKIIα^+^ neurons projection to VM, ventromedial thalamus (A, AP = - 1.67 mm); ACC, anterior cingulate cortex (B, AP = + 0.25 mm); CL, claustrum; AI, Anterior Insular Cortex (C, AP = + 1.21 mm); NAc, nucleus accumbens (D, AP = + 1.21 mm).


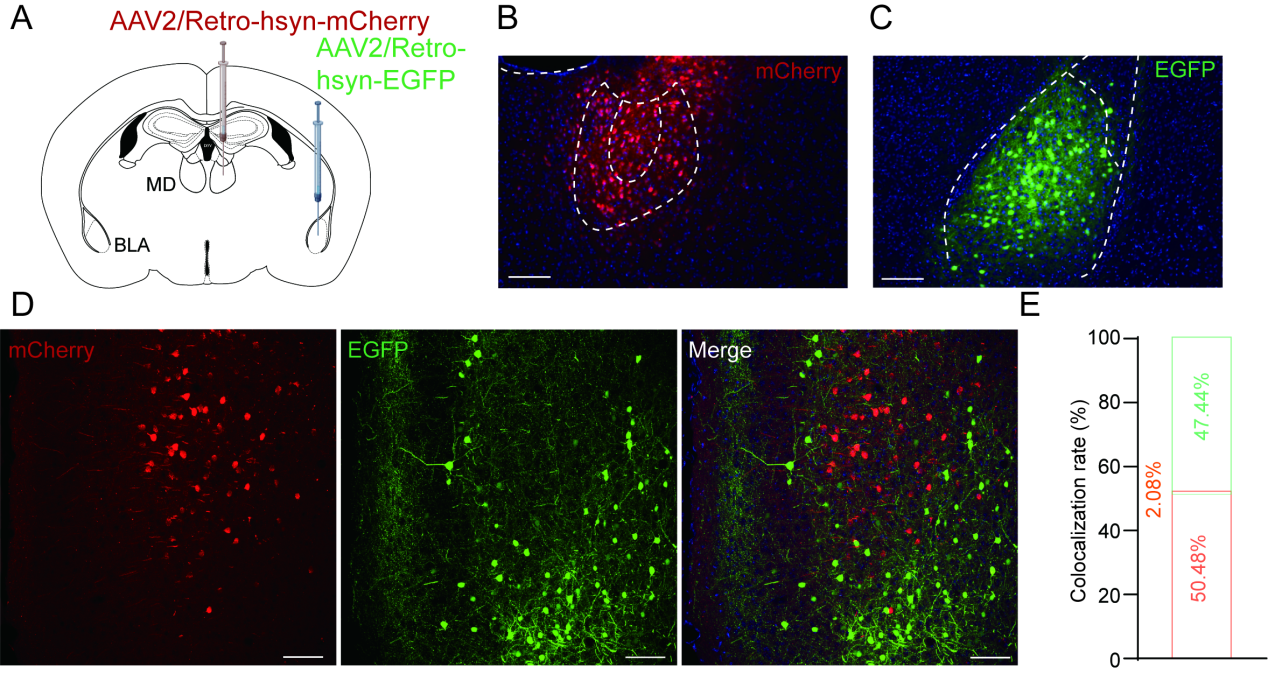


**Figure S4 Result of retrograde tracing approach in C57 mice.**

**(A)** Diagram of retrograde tracing approach in c57 mice. **(B)** Validation of injection site of AAV2/Retro-hsyn-mCherry in MD (AP = - 1.23 mm). **(C)** Validation of injection site of AAV2/Retro-hsyn-EGFP in BLA (AP = - 1.31 mm). Scale bar: 100 μm. **(D)** Representative images of the mOFC neurons projecting to the MD (red) and BLA (green). Scale bar: 50 μm (AP = + 2.30 mm). **(E)** Colocalization of retrograde tracing of MD neurons labeled by mCherry and BLA neurons labeled by EGFP. n = 3 mice.

**
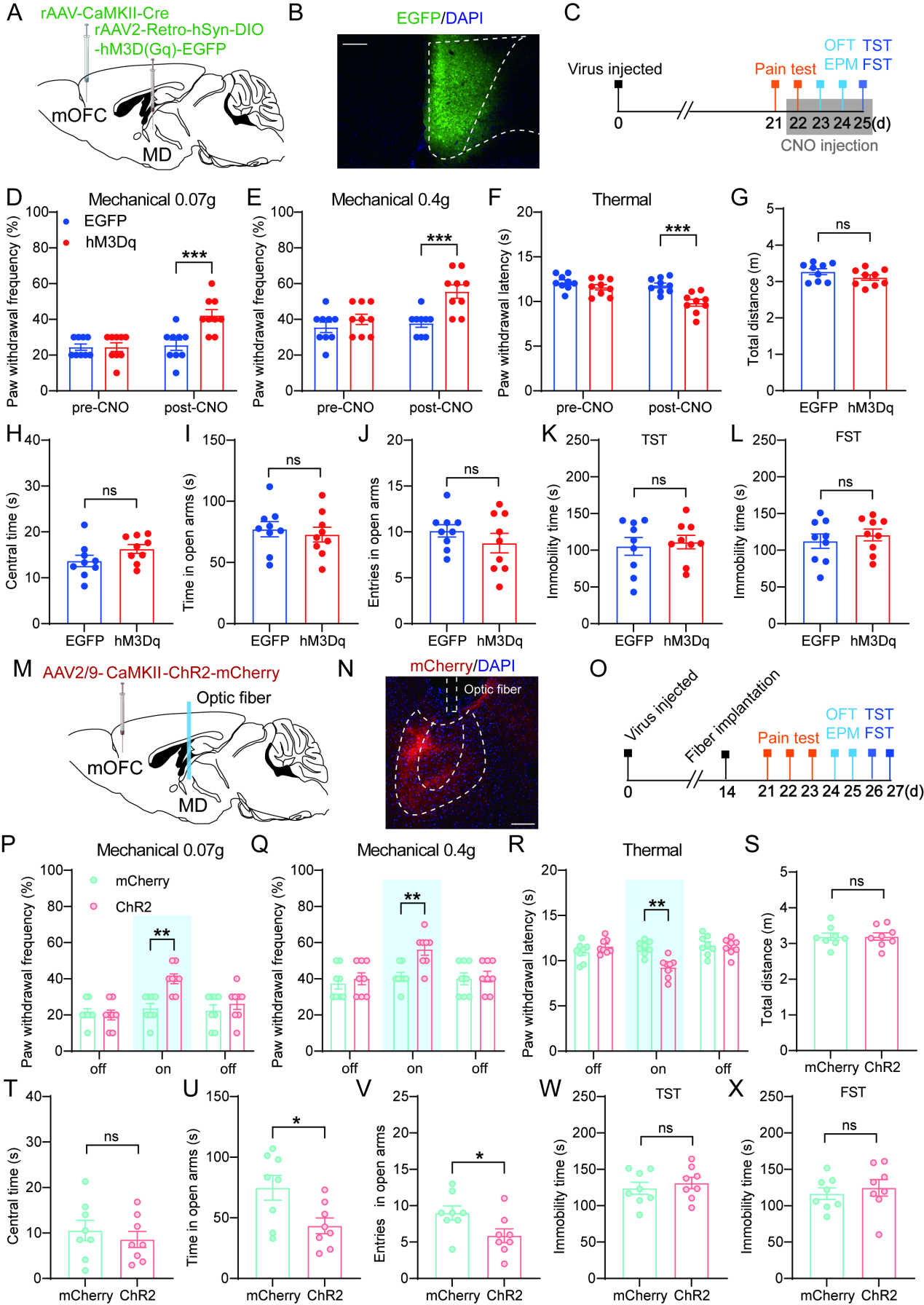
**

**Figure S5 Chemogenetic and optogenetic activation of mOFC^CaMKIIα^-MD projections induces pain behaviors. (A)** Schematic of experimental design. **(B)** The expression of hM3D(Gq)-EGFP in the mOFC (AP = + 2.45 mm). Scale bar: 100 μm. **(C)** Viral injection and behavioral tests timeline. **(D-F)** Effect of chemogenetic activation of mOFC^CaMKIIα^-MD pathway neurons on pain behavior in 0.07g (D), 0.4g (E) von Frey and Hargreaves tests (F) (two-way ANOVA with Sidak post hoc test, n = 9/group). **(G, H)** Quantification of the total distance (G) and central time (H) in the OFT from the EGFP and hM3D(Gq)-EGFP group (unpaired t-test, n = 9/group). **(I, J)** Quantification of the time (I) and entries (J) in open arms in the EPM from the EGFP and hM3D(Gq)-EGFP group (unpaired t-test, n = 9/group). **(K, L)** Immobility time in TST (K) and FST (L) from the EGFP and hM3D(Gq)-EGFP group (unpaired t-test, n = 9/group). **(M)** Schematic of experimental design. **(N)** The axon terminals expression of ChR2-mCherry and optic fiber implantation in the MD (AP = - 1.23 mm). Scale bar: 100 μm. **(O)** Viral injection and behavioral tests timeline. **(P-R)** Effect of optogenetic activation of mOFC^CaMKIIα^-MD pathway neurons on pain behavior in 0.07g (P), 0.4g (Q) von Frey and Hargreaves tests (R) (two-way ANOVA with Sidak post hoc test, n=8/group). **(S, T)** Quantification of the central time (S) and total distance (T) in the OFT from the mCherry and ChR2-mCherry group (unpaired t-test, n = 8/group). **(U-V)** Quantification of the time (U) and entries (V) in open arms of the EPM from the mCherry and ChR2-mCherry group (unpaired t-test, n = 8/group). **(W, X)** Immobility time in TST (W) and FST (X) from the mCherry and ChR2-mCherry group (unpaired t-test, n = 8/group). Data were presented as the mean ± SEM. *P < 0.05, **P <0.01, ***P < 0.001, ns = no significant.


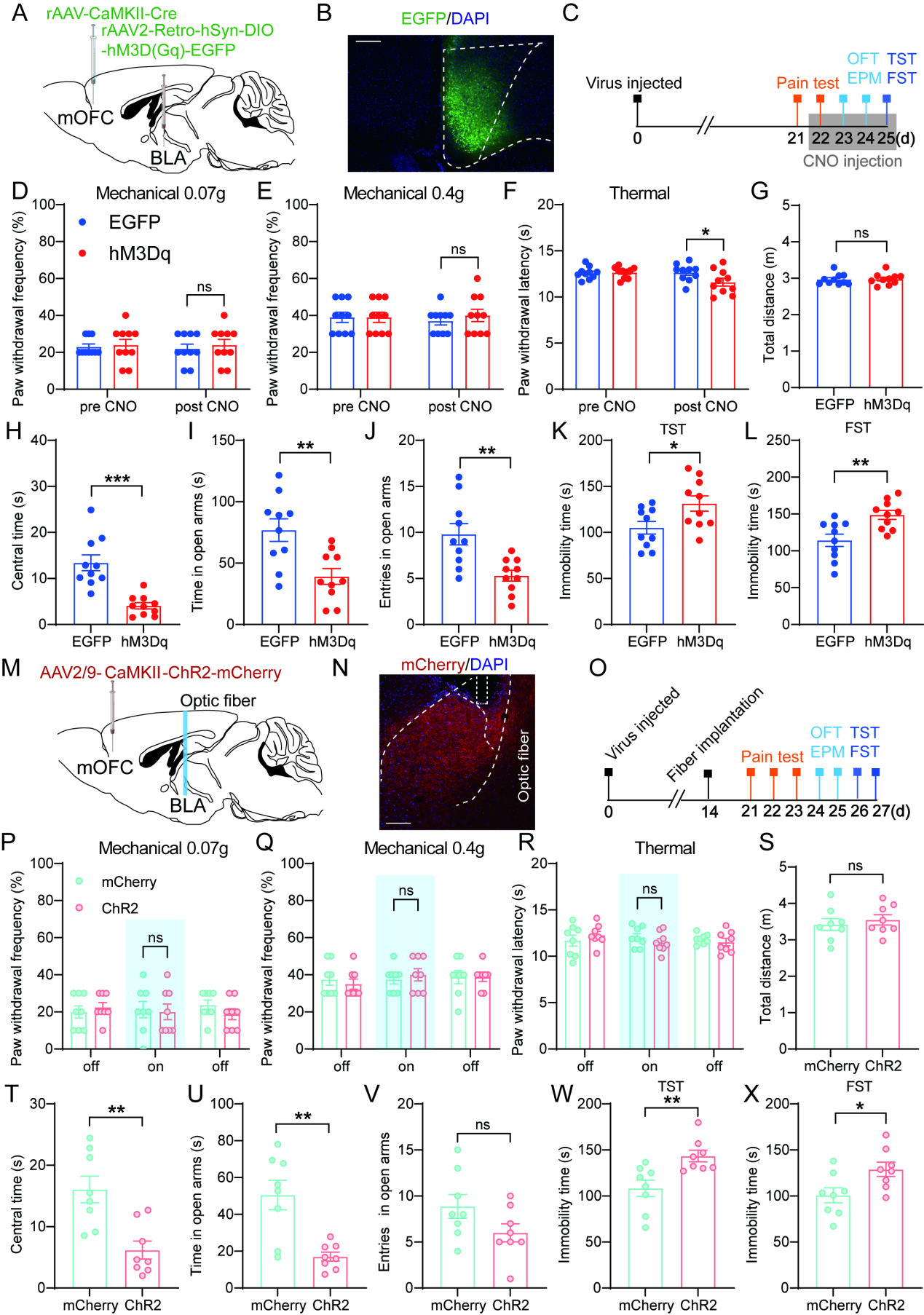


**Figure S6. Chemogenetic and optogenetic activation of mOFC^CaMKIIα^-BLA projections induces anxiodepressive-like behaviors. (A)** Schematic of experimental design. **(B)** The expression of hM3D(Gq)-EGFP in the mOFC (AP = + 2.45 mm). Scale bar: 100 μm. **(C)** Viral injection and behavioral tests timeline. **(D-F)** Effect of chemogenetic activation of mOFC^CaMKIIα^-BLA pathway neurons on pain behavior in 0.07g (D), 0.4g (E) von Frey and Hargreaves tests (F) (two-way ANOVA with Sidak post hoc test, n = 10/group). **(G, H)** Quantification of the total distance (G) and central time (H) in the OFT from the EGFP and hM3D(Gq)-EGFP group (unpaired t-test, n = 10/group). **(I, J)** Quantification of the time (I) and entries (J) in open arms in the EPM from the EGFP and hM3D(Gq)-EGFP group (unpaired t-test, n = 10/group). **(K, L)** Immobility time in TST (K) and FST (L) from the EGFP and hM3D(Gq)-EGFP group (unpaired t-test, n = 10/group). **(M)** Schematic of experimental design. **(N)** The axon terminals expression of ChR2-mCherry and optic fiber implantation in the BLA (AP = - 1.31 mm). Scale bar: 100 μm. **(O)** Viral injection and behavioral tests timeline. **(P-R)** Effect of optogenetic activation of mOFC^CaMKIIα^-BLA pathway neurons on pain behavior in 0.07g (P), 0.4g (Q) von Frey and Hargreaves tests (R) (two-way ANOVA with Sidak post hoc test, n=8/group). **(S, T)** Quantification of total distance (S) and the central time (T) in the OFT from the mCherry and ChR2-mCherry group (unpaired t-test, n = 8/group). **(U-V)** Quantification of the time (U) and entries (V) in open arms of the EPM from the mCherry and ChR2-mCherry group (unpaired t-test, n = 8/group). **(W, X)** Immobility time in TST (W) and FST (X) from the mCherry and ChR2-mCherry group (unpaired t-test, n = 8/group). Data were presented as the mean ± SEM. *P < 0.05, **P <0.01, ***P < 0.001, ns = no significant.
